# The causal effect of *Helicobacter pylori* infection on coronary heart disease is mediated by the body mass index: a Mendelian randomization study

**DOI:** 10.1038/s41598-024-51701-8

**Published:** 2024-01-19

**Authors:** Bing Li, Yaoting Zhang, Yang Zheng, He Cai

**Affiliations:** Department of Cardiovascular Diseases, The First Hospital of Jilin University, Changchun, 130021 Jilin China

**Keywords:** Cardiovascular diseases, Microbiology, Risk factors

## Abstract

The association between *Helicobacter pylori (H. pylori)* infection and coronary heart disease (CHD) remains controversial, with an unclear causal link. This study employed bidirectional Mendelian randomization (MR) method, using *H. pylori* infection as the exposure, to investigate its causal relationship with CHD diagnosis, prognosis, and potential pathogenesis. *H. pylori* infection exhibited a causal association with body mass index (BMI) (β = 0.022; 95% CI 0.008–0.036; *p* = 0.001). Conversely, there was no discernible connection between *H. pylori* infection and the diagnosis of CHD (OR = 0.991; 95% CI 0.904–1.078; *p* = 0.842; IEU database; OR = 1.049; 95% CI 0.980–1.118; *p* = 0.178; FinnGen database) or CHD prognosis (OR = 0.999; 95% CI 0.997–1.001; *p* = 0.391; IEU database; OR = 1.022; 95% CI 0.922–1.123; *p* = 0.663; FinnGen database). Reverse MR analysis showed no causal effect of CHD on *H. pylori* infection. Our findings further support that *H. pylori* infection exerts a causal effect on CHD incidence, mediated by BMI. Consequently, eradicating or preventing *H. pylori* infection may provide an indirect clinical benefit for patients with CHD.

## Introduction

Coronary heart disease (CHD) is caused by atherosclerosis, which includes angina pectoris and myocardial infarction (MI) and is the leading cause of mortality in many countries^1^. The etiology, pathogenesis and prognosis of CHD are complex and have not been fully understood until recently. *Helicobacter pylori* (*H. pylori)* is a gram-negative bacterium that primarily inhabits the stomach and duodenum^2^. More than half of the world's population has been infected with *H. pylori*^3^. In addition to causing gastrointestinal diseases^4^, *H. pylori* can also induce systemic reactions, including abnormal glucose^5^ and lipid metabolism^6^, heightened blood hypercoagulability^7,8^, and chronic inflammatory reactions^9–11^, and is accompanied by vitamin (including vitamin B12, vitamin C, and vitamin D) deficiency^12^. While these reactions represent risk factors for CHD, it remains uncertain whether *H. pylori* influences the occurrence of CHD through these reactions.

However, the relationship between *H. pylori* infection and CHD is still controversial. Several studies have shown that *H. pylori* infection is not significantly related to the occurrence or severity of CHD^13,14^; however, some studies have shown that *H. pylori* infection is one of the main causes of CHD^15,16^. Studies have reported that eradication therapy for *H. pylori* can reduce the levels of peripheral blood inflammatory cytokines, such as interleukin-1β (IL-1β), interleukin-8 (IL-8), and tumor necrosis factor-α (TNF-α) in patients. These inflammatory cytokines are implicated in the development of atherosclerosis and CHD, and their elevation increases the incidence of restenosis in patients after percutaneous transluminal coronary angioplasty (PTCA)^17–19^. The probability of MI in *H. pylori-*infected patients is twice that in uninfected individuals^20^. Another study used infrared radiation spectroscopy to measure the levels of triglycerides, C-reactive protein, homocysteine, low-density lipoprotein (LDL), and TNF-α in peripheral blood. The results showed that, compared with healthy individuals, CHD patients with *H. pylori* infection had elevated triglyceride levels and inflammation^21^. An Asian study also confirmed that *H. pylori* infection can increase the risk of CHD in the next 10 years^22^. At present, the evidence for a link between *H. pylori* infection and CHD is based on observational studies, and there may be some unknown confounding factors that affect judgment of the results. To address this controversial clinical issue, a study that removes confounding factors to accurately determine the causal relationship between *H. pylori* infection and CHD is urgently needed. In addition, although the infection rate of *H. pylori* is relatively high, *H. pylori* infection is not routinely screened, and many infected individuals are unaware of having this infection. Exploring the causal relationship between the two will help determine whether routine screening and treatment of *H. pylori* is one of the prevention and treatment strategies for CHD.

Mendelian randomization (MR) has emerged as a popular epidemiological statistical method that can remove confounding factors and accurately determine the causal relationship between two variables. The method relies on the use of the public genome-wide association study (GWAS) database to obtain instrumental variables (IVs) that are strongly related to exposure but are not related to outcomes or confounding factors. IVs are usually single nucleotide polymorphisms (SNPs), and the causal relationship between exposure and outcomes can be accurately inferred using IVs. In this study, we used *H. pylori* infection as the exposure and applied the bidirectional MR method to infer the relationship between *H. pylori* infection and the diagnosis, prognosis, and possible pathogenesis of CHD. We also used CHD as the exposure to explore the reverse causal relationship between CHD and *H. pylori* infection, two step MR analyses were used to explore indirect pathogenic factors of *H. pylori* infection, with the aim of clarifying this relationship and providing clinical suggestions for the diagnosis and treatment of CHD, providing new insights for CHD.

## Methods

### Study design

**Figure 1:**
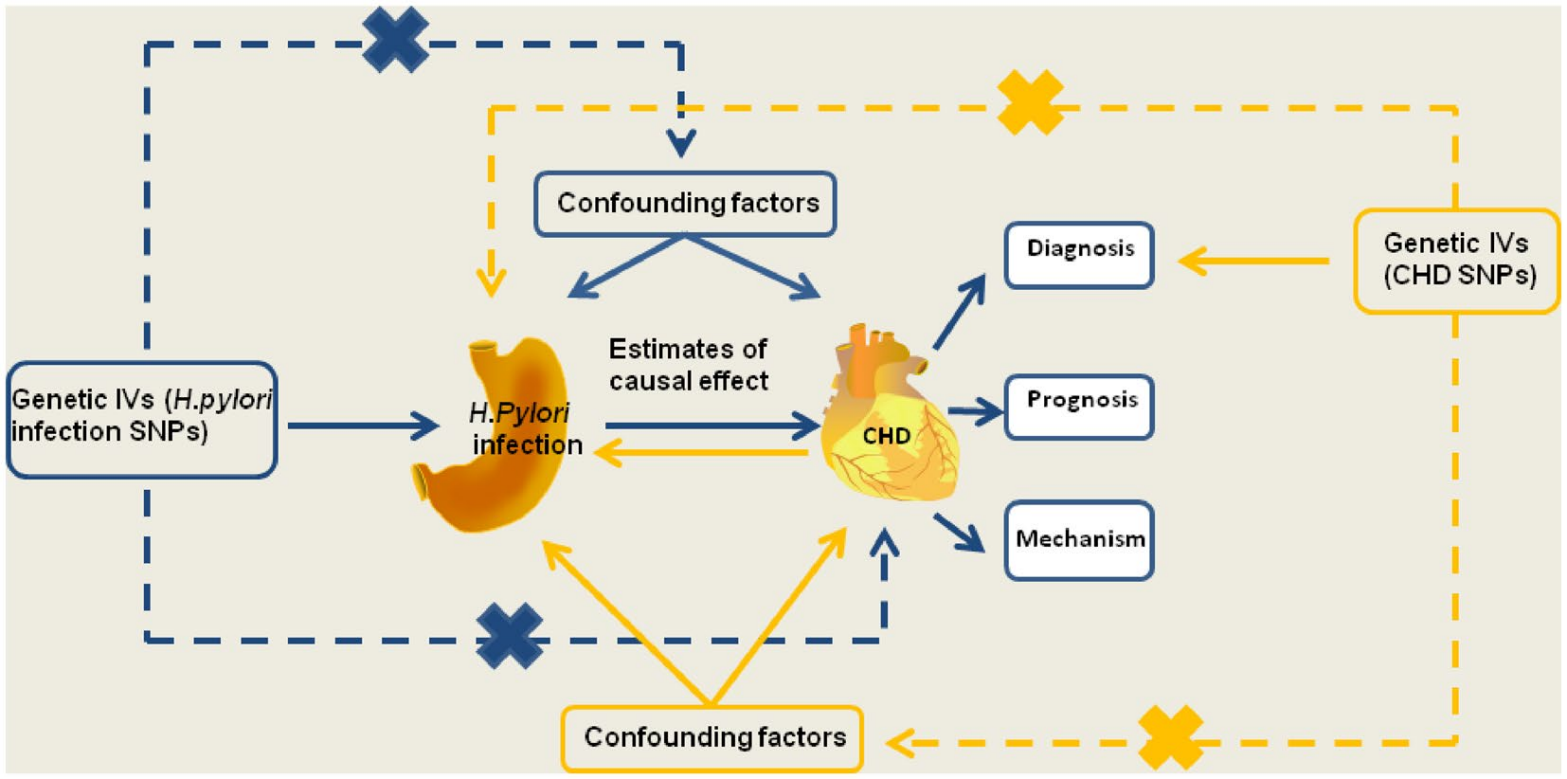
Schematic representation of the MR study on the causal relationship between *H. pylori* infection and CHD incidence. CHD, coronary heart disease; IVs, instrumental variables; *H. pylori*, *Helicobacter pylori*; SNP, single-nucleotide polymorphism.

For the current study, we used IVs as a proxy for exposure, and then conducted an MR analysis to test the association between exposure and outcome^23^. MR is based on three principle assumptions: (1) correlation assumption: IVs are strongly correlated with exposure; (2) exclusivity hypothesis: IVs are not associated with outcomes; and (3) independence hypothesis: IVs are independent of other confounding factors^24^ (Fig. 1).

### Description of the data sources

The genetic association of CHD was derived from the CARDIoGRAMplusC4D Consortium, which included 60,801 cases and 123,504 control subjects from 48 studies, and of which 77% of the participants were of European ancestry and 19% were of South and East Asian ancestry^25^. We also collected summary statistics for CHD, MI and angina pectoris, which were derived from the FinnGen database (https://www.fifinngen.fifi/en)^26^. *H. pylori* infection data were derived from the European Bioinformatics Institute (EBI) database (https://gwas.mrcieu.ac.uk/datasets/ieu-b-4905/) and included 1058 cases and 3625 controls. GWAS data were also collected to investigate the causal effect between *H. pylori* infection and the prognostic data for CHD, including major adverse cardiovascular events (MACE; Neale laboratory and FinnGen database), heart failure (Neale laboratory), heart arrhythmia, heart attack, stroke, target heart rate (HR) reached, and maximum HR data [MRC Integrated Epidemiology Unit (MRC-IEC), https://www.bristol.ac.uk/integrative-epidemiology]. In addition, GWAS data on the possible pathogenesis between *H. pylori* and CHD were also obtained, including fasting blood glucose data from the EBI database, body mass index (BMI) data from the MRC-IEU database, and lipid trait data from the UK Biobank database. Vitamin data were obtained from the MRC-IEU database. Inflammation data were downloaded from the public database IEU (https://gwas.mrcieu.ac.uk/). The GWAS data are detailed in Table 1 and have been approved by the author or the Consortium.

**Table 1:** Details of the studies included in the Mendelian randomization analyses.

Phenotype	Consortium/Author/Database	Ethnicity	Sample size (cases/controls)	Year	SNPs (n)	Web source
*H. polyri* infection	EBI	European	1058/3625	2021	7,247,045	https://gwas.mrcieu.ac.uk/datasets/ieu-b-4905/
CHD	CARDIoGRAMplusC4D	Mixed	60,801/123,504	2015	9,455,779	https://gwas.mrcieu.ac.uk/datasets/ieu-a-7/
CHD	FinnGen study	European	30,952/187,840	2021	16,380,466	https://gwas.mrcieu.ac.uk/datasets/finn-b-I9_ISCHHEART/
MI	FinnGen study	European	12,801/187,840	2021	16,380,433	https://gwas.mrcieu.ac.uk/datasets/finn-b-I9_MI/
Angina pectoris	FinnGen study	European	18,168/187,840	2021	16,380,426	https://gwas.mrcieu.ac.uk/datasets/finn-b-I9_ANGINA/
FBG	EBI	European	58,074 participants	2012	2,599,409	https://gwas.mrcieu.ac.uk/datasets/ebi-a-GCST005186/
TG	UK Biobank	European	441,016 participants	2020	12,321,875	https://gwas.mrcieu.ac.uk/datasets/ieu-b-111/
HDL	UK Biobank	European	403,943 participants	2020	12,321,875	https://gwas.mrcieu.ac.uk/datasets/ieu-b-109/
LDL	UK Biobank	European	440,546 participants	2020	12,321,875	https://gwas.mrcieu.ac.uk/datasets/ieu-b-110/
BMI	MRC-IEU	European	454,884 participants	2018	9,851,867	https://gwas.mrcieu.ac.uk/datasets/ukb-b-2303/
Vitamin C	MRC-IEU	European	39,880/420,471	2018	9,851,867	https://gwas.mrcieu.ac.uk/datasets/ukb-b-15175/
Vitamin D	MRC-IEU	European	17,879/442,472	2018	9,851,867	https://gwas.mrcieu.ac.uk/datasets/ukb-b-12648/
Vitamin B12	MRC-IEU	European	64,979 participants	2018	9,851,867	https://gwas.mrcieu.ac.uk/datasets/ukb-b-19524/
Interleukin-18	Folkersen L	European	21,758 participants	2020	13,102,515	https://gwas.mrcieu.ac.uk/datasets/ebi-a-GCST90012024/
Interleukin-6	Folkersen L	European	21,758 participants	2020	11,782,139	https://gwas.mrcieu.ac.uk/datasets/ebi-a-GCST90012005/
Interleukin-8	Folkersen L	European	21,758 participants	2020	12,717,989	https://gwas.mrcieu.ac.uk/datasets/ebi-a-GCST90011994/
Interleukin-4	Ahola-Olli AV	European	8124 participants	2016	9,786,064	https://gwas.mrcieu.ac.uk/datasets/ebi-a-GCST004453/
Interleukin-10	Ahola-Olli AV	European	7681 participants	2016	9,793,415	https://gwas.mrcieu.ac.uk/datasets/ebi-a-GCST004444/
TNF-α	Ahola-Olli AV	European	3454 participants	2016	9,500,449	https://gwas.mrcieu.ac.uk/datasets/ebi-a-GCST004426/
MACEs	Neale lab	European	10,157/351,037	2018	13,295,130	https://gwas.mrcieu.ac.uk/datasets/ukb-d-I9_CHD/
MACEs	FinnGen study	European	21,012/197,780	2021	16,380,466	https://gwas.mrcieu.ac.uk/datasets/finn-b-I9_CHD/
Death	FinnGen study	European	7563/211,229	2021	16,380,466	https://gwas.mrcieu.ac.uk/datasets/finn-b-I9_K_CARDIAC/
Heart arrhythmia	MRC-IEU	European	2545/460,388	2018	9,851,867	https://gwas.mrcieu.ac.uk/datasets/ukb-b-3703/
Heart attack	MRC-IEU	European	10,693/451,187	2018	9,851,867	https://gwas.mrcieu.ac.uk/datasets/ukb-b-11590/
Stroke	MRC-IEU	European	7055/454,825	2018	9,851,867	https://gwas.mrcieu.ac.uk/datasets/ukb-b-8714/
Heart failure	Neale lab	European	1405/359,789	2018	9,858,439	https://gwas.mrcieu.ac.uk/datasets/ukb-d-I9_HEARTFAIL/
Target HR	MRC-IEU	European	6995/61,431	2018	9,851,867	https://gwas.mrcieu.ac.uk/datasets/ukb-b-16609/
Maximum HR	MRC-IEU	European	68,409 participants	2018	9,851,867	https://gwas.mrcieu.ac.uk/datasets/ukb-b-14461/

*BMI* body mass index, *CHD* coronary heart disease, *CRP* C-reactive protein, *FBG* fasting blood glucose, *HDL-C* high-density lipoprotein cholesterol, *LDL-C* low-density lipoprotein cholesterol, *MACEs* major adverse cardiovascular events, *Maximum HR* maximum heart rate during fitness test, *MI* myocardial infarction, *Target HR* target heart rate achieved, *TG* triglycerides, *TNF-α* tumor necrosis factor-α.

The demographic characteristics of GWAS data for *H. pylori* infection are as follows: pregnant women residing in Avon, UK, with expected delivery dates between April 1, 1991, and December 31, 1992, were invited to participate in the ALSPAC study. The overall sample size for analyses, incorporating data collected after the age of seven, was determined. Serum antibody levels related to *H. pylori* infection were measured using ELISA, ultimately providing GWAS data associated with *H. pylori*^27^. In the FinnGen database, the average age of GWAS data is 63 years, with a male proportion of 43.5% (source: https://www.nature.com/articles/s41586-022-05473-8). For the UKB database, the average age of GWAS data is 56.9 years, with a male proportion of 45.8%^28^. The remaining GWAS datasets may have been obtained through meta-analysis, making it challenging to acquire information on gender and age.

### Selection of genetic IVs for *H. pylori* Infection

The genetic IVs were acquired from previous literature^29–31^. This study involved bidirectional MR analysis of *H. pylori* infection and non-alcoholic fatty liver disease. The SNPs rs368433 and rs10004195, located in the Toll-like receptor 10 (*TLR10*) gene (4p14) and the Fc gamma RIIA (*FCGR2A*) gene (1q23.3), respectively, have been reported to be strongly associated with *H. pylori* infection and are used as IVs^29^. Instrument strength was evaluated using the F-statistic for each allele, and if the F-statistic was greater than 10, it was considered that the potential weak instrument bias was minimized^30,31^. The F-statistic for each SNP was derived from the following equation:

$$ \begin{aligned} & {\text{F}} = {\text{R}}^{2} \left( {{\text{N}} - 2} \right){/}\left( {1 - {\text{R}}^{2} } \right) \\ & {\text{R}}^{2} = 2 \times \left( {1 - {\text{MAF}}} \right) \times {\text{MAF}} \times \beta^{2} \\ \end{aligned} $$

where R^2^ is the proportion of variation explained by IVs, N is the sample size of the exposure dataset, and MAF indicates the minor allele frequency. In our study, all F-statistics were greater than 100 and, therefore, suitable for our analysis (Supplementary Table S1).

### Selection of genetic IVs for CHD and BMI

The genetic IVs for CHD and the potential pathogenesis of *H. pylori* infection were obtained from the GWAS summary statistics. The following three steps were subsequently used to screen for strong correlations with CHD but not with *H. pylori* infection or confounding factors to ensure that the effect of each allele (containing each SNP) was the same. First, SNPs strongly related to exposure were screened (*p* < 5 × 10^˗8^). Second, independence was set to remove linkage disequilibrium (LD; r^2^ < 0.001, window size = 10,000 kb, *p* < 5 × 10^˗8^) and calculate the statistical strength (F-statistical > 10). Third, the exposure and outcome datasets were harmonized to ensure that the effect alleles belonged to the same allele. The SNPs screened by these strict procedures can be used as IVs for subsequent analysis (Supplementary Table S2). The genetic IVs for BMI were obtained by the same screening method (Supplementary Table S3).

### Statistical analysis and data visualization

All analyses were performed using R programming software (R4.1.2, https://www.rproject.org/). The primary MR analysis was conducted using the Wald ratio and the inverse variance weighting (IVW) method, and a two-sided *p*-value < 0.05 was considered indicative of statistical significance. Due to the multiple comparisons, we further applied a Bonferroni corrected threshold for statistical significance (0.05/number of analyses)^32^ (Table 2). In reverse MR analysis and two step MR analysis, because of the large number of IVs, we applied two complementary methods (MR‒Egger and weighted-median) to increase the stability of the results. MR analyses were performed using the R-based package “TwoSampleMR” (version 0.5.6). Forest plots were generated using the “ggplot2” R package (version 3.4.0).

**Table 2:** The Bonferroni corrected threshold for statistical significance in the diagnosis, prognosis, and pathogenesis of CHD.

Outcome	Number of analyses	Bonferroni corrected threshold
Diagnosis	4	0.0125
Prognosis	9	0.0056
Pathogenesis	14	0.0036

## Results

### Causal effect of *H. pylori* infection on the diagnosis of CHD

**Figure 2:**
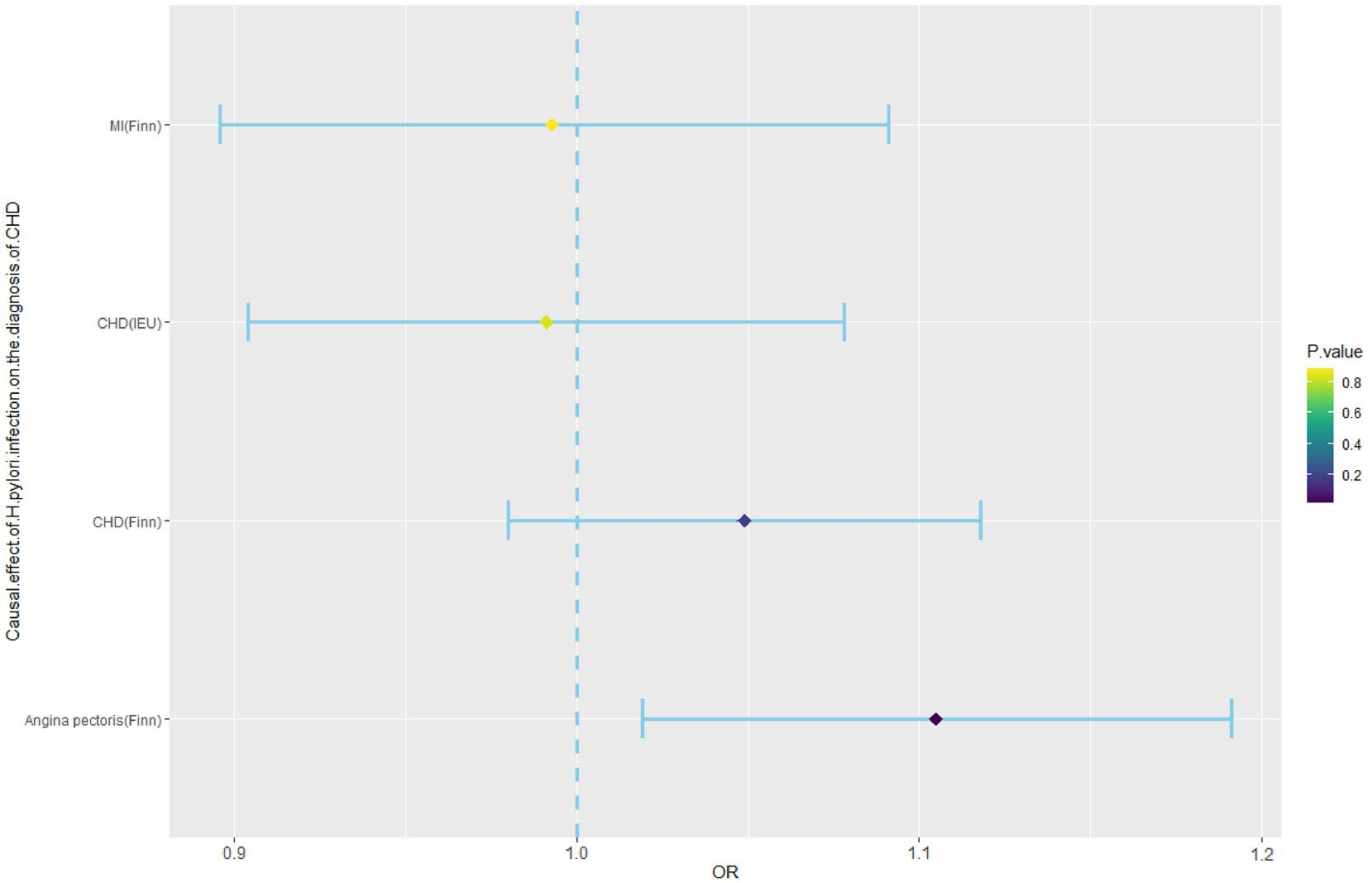
Mendelian randomization results for the effect of *H. pylori* infection on the diagnosis of CHD. CHD, coronary heart disease; *H. pylori*, *Helicobacter pylori*; MI, myocardial infarction; OR, odds ratio.

According to previous studies, the SNPs rs10004195 (T>A) and rs368433 (T>C) are strongly related to *H. pylori* infection^30^. Conventional IVs typically consist of two or more. Although there were only two IVs in this study, these two SNP loci were strongly correlated with *H. pylori* infection, with F values greater than 100, and their efficacy was more than 10 times that of conventional IVs (Supplementary Table S1). The two corresponding genes are TLR10 and FCGR2A. TLR10 is a key gene that regulates the release of inflammatory factors during *H. pylori* infection^33^, and FCGR2A is also a key gene that regulates the intestinal^34^ and cardiac inflammatory responses^35^. We therefore used these two SNPs as IVs of *H. pylori* infection to predict the relationship between *H. pylori* infection and the diagnosis of CHD, MI, or angina pectoris^29^. *H. pylori* infection was not associated with the occurrence of CHD (IEU) [odds ratio (OR), 0.991; 95% confidence interval (CI) 0.904–1.078; *p*-value = 0.842], CHD (Finn) (OR, 1.049; 95% CI 0.980–1.118; *p*-value = 0.178), angina pectoris (OR, 1.105; 95% CI 1.019–1.191;* p*-value = 0.023), or MI (OR, 0.993; 95% CI 0.896–1.091; *p*-value = 0.889) according to the IVW method. (Fig. 2, Supplementary Table S4). Although the causal analysis between *H. pylori* infection and angina pectoris showed a *p* value < 0.05, it is imperative to consider that this study included four distinct outcomes, each subjected to separate analyses. In accordance with the Bonferroni threshold correction method, the adjusted significance level dictates that the effective p value should be < 0.0125 to account for the multiple comparisons conducted (Table 2). Therefore, our analysis revealed that there is no causal relationship between *H. pylori* infection and CHD diagnosis.

### Causal effect of *H. pylori* infection on the prognosis of CHD

**Figure 3:**
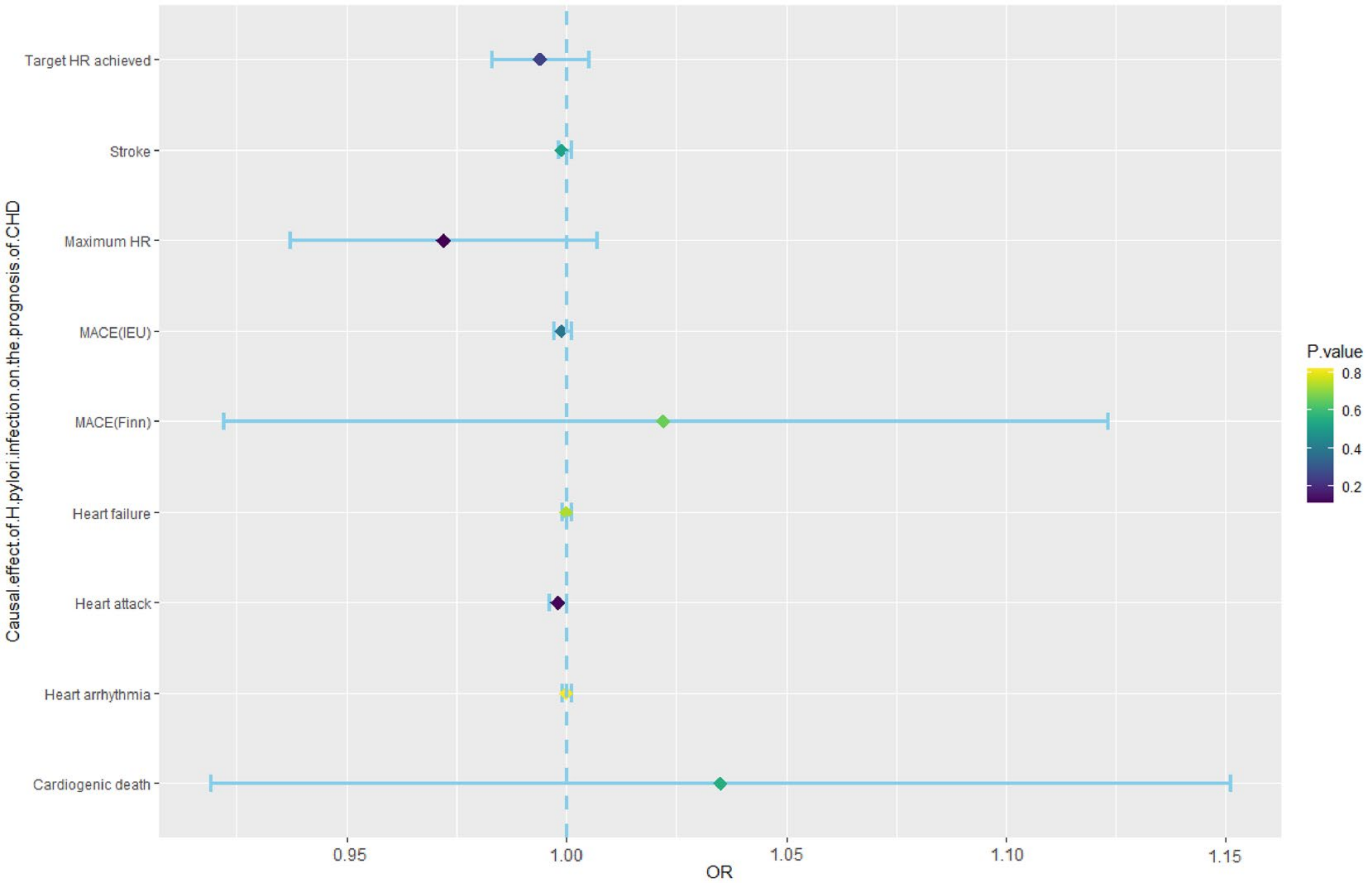
Mendelian randomization results for the effect of *H. pylori* infection on the prognosis of CHD patients. CHD, coronary heart disease; *H. pylori*, *Helicobacter pylori*; MACEs, major adverse cardiovascular events; Maximum HR, maximum heart rate during fitness test; OR, odds ratio; Target HR achieved, reached target heart rate.

MR analyses were further performed to examine the causal association between *H. pylori* infection and the prognosis of CHD, including MACE, heart arrhythmia, heart attack, stroke, heart failure, target HR achieved, and maximum HR during the fitness test. The incidence of MACE, which mainly includes heart arrhythmia, heart attack, stroke, and heart failure, is currently the main method for determining the prognosis of CVD patients. In addition, the maximum HR and target HR in cardiopulmonary exercise tests can also predict the prognosis of CHD patients and are negatively correlated with their prognosis^36,37^. Therefore, this study used the above factors as prognostic indicators for CHD. The analysis showed that *H. pylori* infection had no causal effect on MACE (OR, 0.999; 95% CI 0.997–1.001; *p*-value = 0.391; IEU database; OR, 1.022; 95% CI 0.922–1.123; *p*-value = 0.663; FinnGen database), heart arrhythmia (OR, 1.000; 95% CI 0.999–1.001; *p*-value = 0.823), heart attack (OR, 0.998; 95% CI 0.996–1.000; *p*-value = 0.124), stroke (OR, 0.999; 95% CI 0.998–1.001; *p*-value = 0.525), heart failure (OR, 1.000; 95% CI 0.999–1.001; *p*-value = 0.741), target HR achieved (OR, 0.994; 95% CI 0.983–1.005; *p*-value = 0.252), or maximum HR (OR, 0.972; 95% CI 0.937–1.007; *p*-value = 0.115) (Fig. 3, Supplementary Table S5).

### Causal effect of *H. pylori* infection on the pathogenesis of CHD

**Figure 4:**
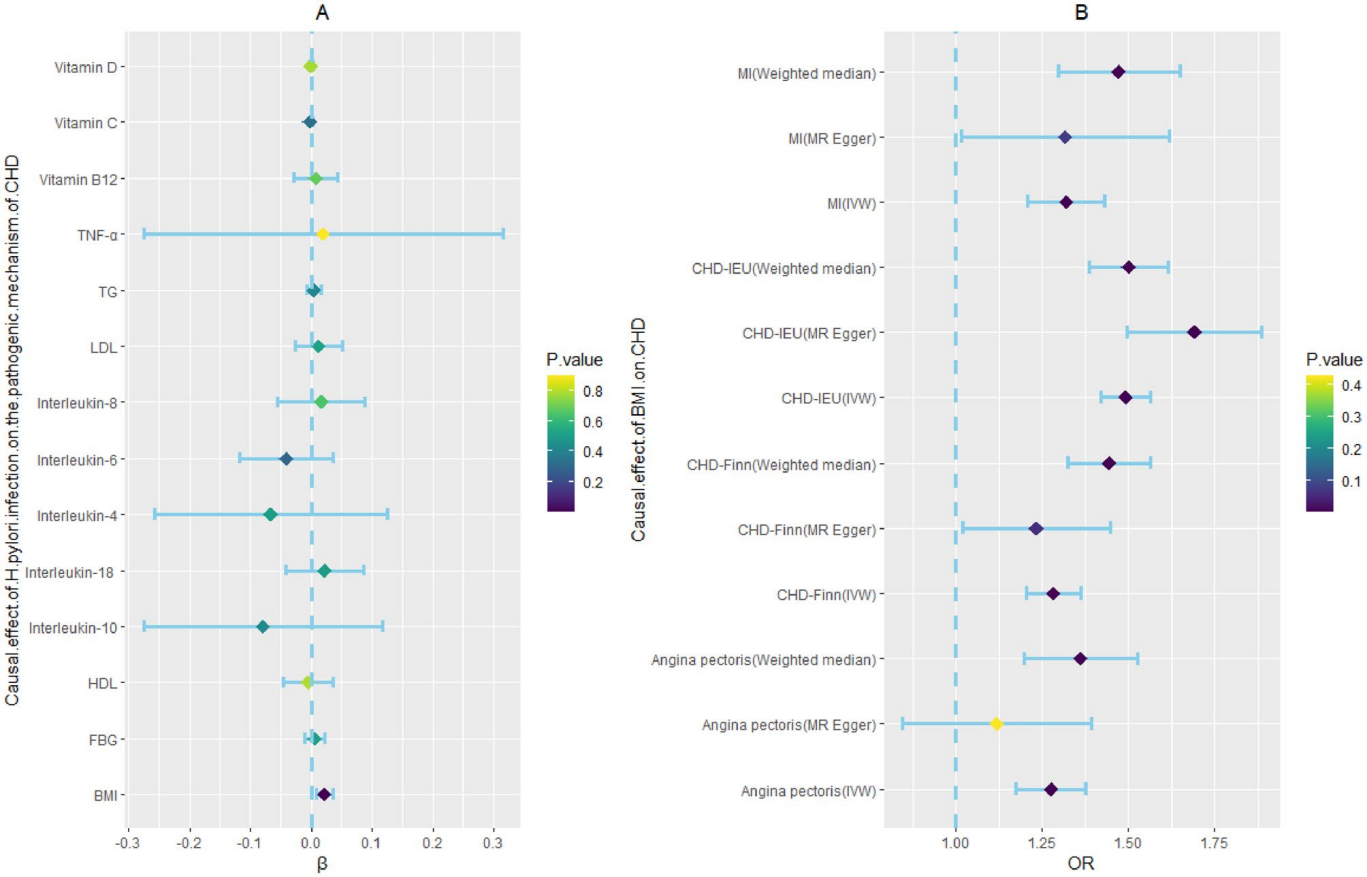
Two step Mendelian randomization results for the effect of *H. pylori* infection on CHD incidence (the pathogenic mechanism of CHD). BMI: body mass index; CRP, C-reactive protein; FBG, fasting blood glucose; *H. pylori*, *Helicobacter pylori*; HDL-C, high-density lipoprotein cholesterol; LDL-C, low-density lipoprotein cholesterol; OR, odds ratio. TG, triglyceride; TNF-α, tumor necrosis factor-α. Dark blue dots represent significant differences as indicated by the *P* values.

Based on previous research, we summarized the pathogenic mechanisms of *H. pylori* infection on CHD, and these included abnormal glucose and lipid metabolism, vitamin deficiency (including vitamin B12, vitamin C, and vitamin D), and chronic inflammatory reactions. In addition to metabolic abnormalities and chronic inflammation, vitamin deficiency has also been found to be associated with the occurrence and development of CHD. *H. pylori* infection can cause damage to gastric wall cells, leading to a decrease in the secretion of endogenous factors by gastric wall cells and a decrease in the absorption of vitamin B12 in the small intestine. Moreover, deficiencies in vitamin C and vitamin D, both of which are associated with the *H. pylori* infection progression, represent risk factors for CHD. Therefore, we used these factors as indicators of CHD pathogenesis^38,39^. To explore the causal relationship between *H. pylori* infection and CHD pathogenesis, we used *H. pylori* infection as the exposure and pathogenesis as the outcome for MR analysis. According to the MR analyses of abnormal glucose and lipid metabolism, *H. pylori* infection had no association with fasting blood glucose levels (β, 0.006; 95% CI − 0.011 to 0.023; *p*-value = 0.511), triglyceride (TG) levels (β, 0.005; 95% CI − 0.006 to 0.016; *p*-value = 0.409), high-density lipoprotein cholesterol (HDL-C) levels (β, − 0.006; 95% CI − 0.047 to 0.035; *p*-value = 0.788), or low-density lipoprotein cholesterol (LDL-C) levels (β, 0.013; 95% CI − 0.026 to 0.051; *p*-value = 0.515). In the vitamin deficiency MR analysis, we obtained negative results for water-soluble vitamins, including vitamin C (β, − 0.002; 95% CI − 0.006 to 0.002; *p*-value = 0.318) and vitamin B12 (β, 0.008; 95% CI − 0.029 to 0.044; *p*-value = 0.685). The same result was also observed for the fat-soluble vitamins of vitamin D (β, − 0.0003; 95% CI − 0.003 to 0.002; *p*-value = 0.775). In addition, we also analyzed whether *H. pylori* infection contributed to the occurrence of CHD through inflammatory mechanisms and found no causal relationships between *H. pylori* infection and IL-4 (β, − 0.066; 95% CI − 0.258 to 0.125; *p*-value = 0.497), IL-6 (β, − 0.041; 95% CI − 0.117 to 0.035; *p*-value = 0.294), IL-8 (β, 0.017; 95% CI − 0.055 to 0.088; *p*-value = 0.645), IL-10 (β, − 0.079; 95% CI − 0.276 to 0.117; *p*-value = 0.429), IL-18 (β, 0.022; 95% CI − 0.041 to 0.086; *p*-value = 0.493) or TNF-α (β, 0.020; 95% CI − 0.275 to 0.316; *p*-value = 0.893). However, there was a significant causal relationship between *H. pylori* infection and BMI (β, 0.022; 95% CI 0.008–0.036; *p*-value = 0.001), and there was a causal relationship between BMI and CHD incidence (Fig. 4, Supplementary Tables S6, and S7). A study showed that, compared to those in the control group, patients infected with *H. pylori* had increased growth hormone levels and decreased obesity, which promoted appetite increase^40^. Another study suggested that *H. pylori* can affect appetite and dietary habits through the brain-gut axis^41^. Therefore, we speculate that the mechanism by which *H. pylori* promotes an increase in BMI is through the brain-gut axis to alter appetite and promote energy intake.

### Reverse causal effect of CHD on *H. pylori* infection

**Figure 5:**
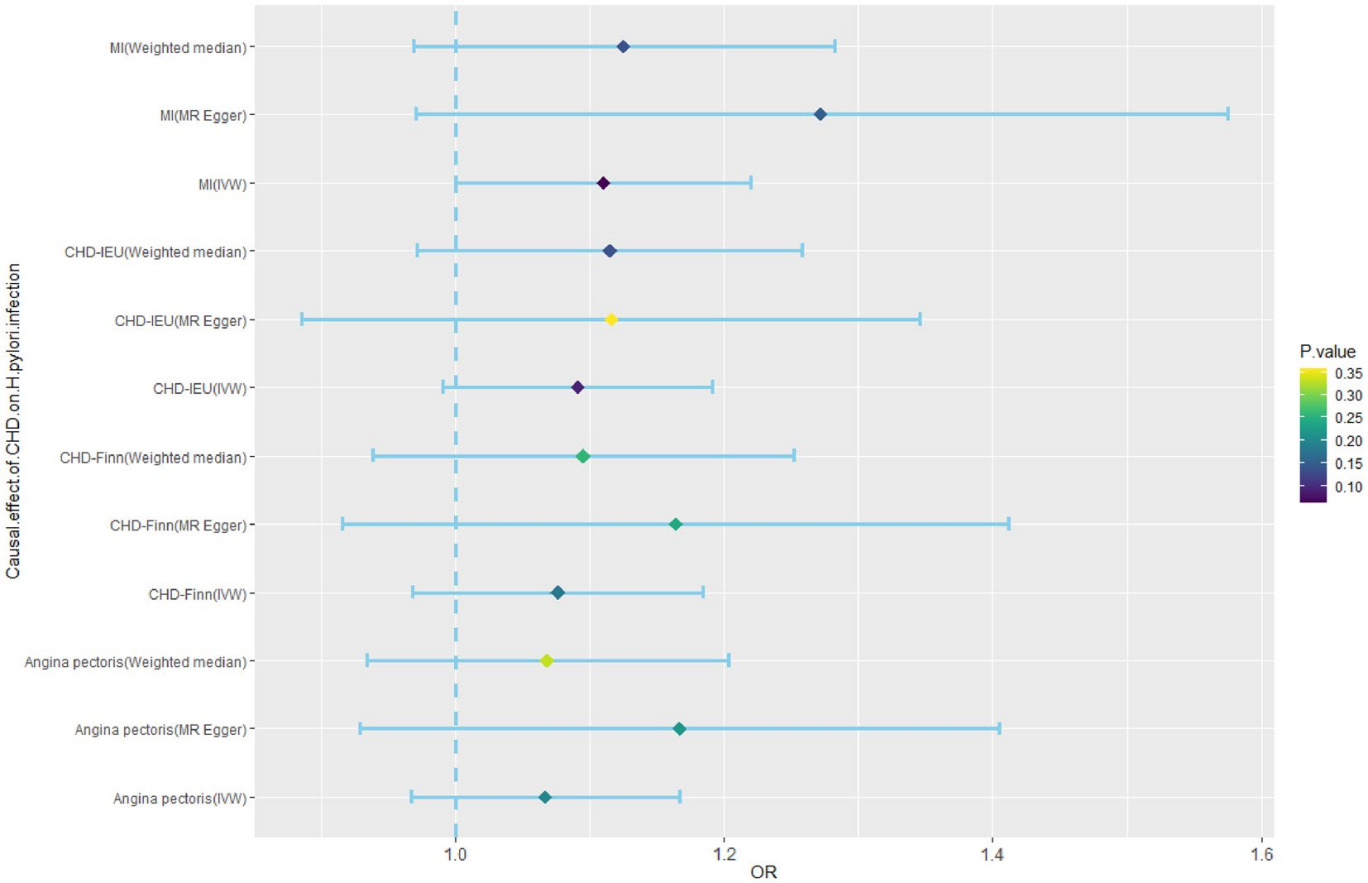
Mendelian randomization results for the effect of CHD on *H. pylori* infection. CHD, coronary heart disease; *H. pylori*, *Helicobacter pylori*; MI, myocardial infarction; OR, odds ratio.

The IVs of CHD, MI, and angina pectoris were identified from public GWAS summary data. Three MR analysis methods, namely, IVW, weighted median, and MR‒Egger, were used for this analysis. None of the three methods had a significant causal effect on *H. pylori* infection (Fig. 5, Supplementary Table S8).

## Discussion

In this study, we used large-scale public GWAS data to analyze the causal relationship between *H. pylori* infection and the risk of CHD using the MR method. The causal effect of *H. pylori* infection on CHD incidence was mediated by BMI.

The association between *H. pylori* infection and CHD is currently controversial. Several studies have reported that *H. pylori* infection is related to the occurrence and prognosis of CHD^19,42,43^. A prospective study revealed that *H. pylori*-infected patients had an increased occurrence of CHD^44^ and adverse events^42^. According to other studies, MI patients infected with *H. pylori* have a higher mortality rate^45^, and the probability of restenosis after PTCA is higher^17^. It has also been shown that MI has a reverse causal effect on *H. pylori*. Young people with MI have twice the probability of *H. pylori* infection as healthy individuals^46,47^. However, some studies have been unable to detect a correlation between the occurrence and development of CHD and *H. pylori* infection, especially among older individuals^48^. A prospective study with a small sample^14^ and meta-analyses of five large samples^14,49^ have provided evidence that *H. pylori* infection is not significantly related to the severity or prognosis of CHD. A prospective study involving 180 patients who underwent stent implantation in a native coronary artery revealed that there is no significant association between *H. pylori* infection and restenosis following PTCA^50^. However, the pathogenic link between *H. pylori* infection and CHD remains controversial. First, in terms of metabolism, the influence of *H. pylori* infection on glucose and lipid metabolism and BMI is controversial. Regarding lipids, a study showed that *H. pylori* infection can reduce the level of HDL and increase the levels of LDL and TG^51^. However, other studies have presented opposite findings^52,53^. Meta-analyses and prospective studies of large samples have shown that eradication of *H. pylori* infection has no significant effect on the levels of HDL, TG, or LDL^6,54^. In terms of glucose metabolism, evidence suggests that *H. pylori* infection may participate in the onset of diabetes and impaired glucose control in diabetes patients^55,56^. Infection with *H. pylori* can increase insulin resistance in both young people and diabetes patients^57^. One study revealed that, compared with that in the control group, the improvement in glucose homeostasis in diabetes patients after successful eradication of *H. pylori* infection was not statistically significant^58^. In terms of body weight, the eradication of *H. pylori* infection has been associated with increased weight in children^59^ and has variable effects on weight in adults—either increasing^60^ or decreasing^61^ it. Additionally, there is a higher observed incidence of *H. pylori* infection among obese individuals^62^. Second, *H. pylori* infection induces alterations in the gastrointestinal microenvironment, potentially impeding the absorption of nutrients, resulting in a deficiency of micronutrients^63^. Poor vitamin B12 absorption has been shown to be related to *H. pylori* infection^64^. The levels of vitamin C and vitamin D are closely related to CHD incidence^65^, but the relationship between vitamins and *H. pylori* infection remains to be confirmed. Third, *H. pylori* can cause an inflammatory reaction. Chronic inflammation caused by *H. pylori* may have dual effects. On the one hand, low-grade inflammation is a common feature of obesity, diabetes, insulin resistance, and dyslipidemia, and *H. pylori* may cause a chronic inflammatory reaction through abnormal metabolism^66^. On the other hand, *H. pylori* causes damage to the gastrointestinal tract^67^, stimulating an increase in interleukin levels^10,11^. *H. pylori* infection has been associated with elevated levels of TNF-α and IL-6 in patients with CHD^68–71^. Conflicting data have also been reported regarding inflammation^72^. The factors involved in the pathogenesis of *H. pylori* infection, which include glucose and lipid metabolism, vitamin deficiency, and chronic inflammatory reactions, are all causes of CHD.

The discrepancy between *H. pylori* infection and CHD could be attributed to multiple factors, such as differences in the race and age of the selected sample population, the small sample size, the low incidence of MACE, the detection method for *H. pylori* infection, and the different follow-up times. These confounding factors may lead to the poor statistical efficiency of the data and may affect the reliability of the experimental results.

This study revealed that *H. pylori* infection has no direct causal effect on the diagnosis or prognosis of CHD. According to our analysis of pathogenesis, *H. pylori* infection has a causal effect on BMI, and BMI has a causal effect on CHD incidence. Therefore, the causal effect of *H. pylori* infection on CHD incidence is mediated by BMI. However, *H. pylori* infection has no causal effect on inflammatory factors (IL-4, IL-6, IL-8, IL-10, IL-18, or TNF-α), vitamins (vitamin B12, vitamin C, or vitamin D), or glucose and lipid metabolism, and there is no reverse causal effect of CHD on *H. pylori* infection.

This study used the MR method to reveal a bidirectional causal relationship between *H. pylori* infection and CHD for the first time and could increase the recognition of pathogenic factors of CHD from the perspective of systems biology. The advantage of MR studies is that the sample size is large, and they involve a natural randomized controlled trial, which eliminates confounding factors. However, this study has several limitations. The GWAS of *H. pylori* infection was based on serological samples, which may not be truly representative of *H. pylori* infection. Furthermore, the samples were obtained from individuals of European ancestry and therefore may not be representative of all populations worldwide. Finally, the screening of IVs in this study was strict, which may have led to negative results.

## Conclusions

Our findings confirm that the causal effect of *H. pylori* infection on CHD incidence is mediated by BMI. Therefore, the eradication or prevention of *H. pylori* infection may indirectly benefit patients with CHD indirectly in the clinic.

### Supplementary Information

Supplementary Tables.

## Data Availability

All data generated or analysed during the study is included in this published article. The datasets for this study are shown in Table 1.
