# Supplementary material for: The causal effect of Helicobacter pylori infection on coronary heart disease is mediated by the body mass index: a Mendelian randomization study
Source: Sci Rep. 2024 Jan 19;14:1688. doi: 10.1038/s41598-024-51701-8 (PMC10798959; doi:10.1038/s41598-024-51701-8)
Supplement: Supplementary file 1 — Supplementary Tables. [file 41598_2024_51701_MOESM1_ESM.pdf]

Supplementary Information

The causal effect of *Helicobacter pylori* infection on coronary heart disease is mediated by the body mass index: a Mendelian randomization study

Bing Li,[1] Yaoting Zhang,[1] Yang Zheng,[*#1] and He Cai,[*#1]

[1]Department of Cardiovascular Diseases, The First Hospital of Jilin University, Changchun, 130021 Jilin China

[#]Contributed equally.

[*]Corresponding author.

Supplementary Table S1. Instrumental SNPs of *H. pylori* infection and F statistics.

Instrumental SNP	Gene	Effect Allele	Other Allele	EAF	BETA	SE	*P*	Sample size (n)	R^2	F
rs368433	FCGR2A	C	T	0.160	0.315	0.056	2E-08	4683	0.027	128.113
rs10004195	TLR10	A	T	0.250	0.358	0.040	1.00E-18	4680	0.048	235.883

Supplementary Table S2. Instrumental SNPs of CHD.

Exposure	SNP	Effect Allele	Other Allele	Exposure							Outcome (*H.pylori*)			
				BETA	EAF	SE	*P*	Sample size (n)	R^2	F	BETA	EAF	SE	*P*
**CHD (IEU)**	rs10080815	G	T	0.247	0.028	0.031	1.33E-15	184305	0.003	602.800	0.047	0.019	0.088	0.595
	rs10139550	G	C	0.055	0.423	0.010	1.38E-08	184305	0.001	276.340	-0.020	0.419	0.024	0.416
	rs10840293	A	G	0.055	0.550	0.010	1.28E-08	184305	0.001	273.533	0.016	0.442	0.024	0.493
	rs11065979	T	C	0.069	0.365	0.011	1.93E-10	184305	0.002	402.643	0.015	0.428	0.024	0.543
	rs11191416	G	T	-0.079	0.127	0.014	4.65E-09	184305	0.001	257.837	0.010	0.083	0.042	0.820
	rs11556924	T	C	-0.073	0.313	0.011	5.34E-11	184305	0.002	418.597	-0.002	0.394	0.024	0.935
	rs115654617	A	C	0.138	0.107	0.016	3.12E-18	184305	0.004	671.475	0.055	0.129	0.035	0.115
	rs11838776	A	G	0.069	0.263	0.011	1.83E-10	184305	0.002	336.736	0.021	0.273	0.026	0.426
	rs1199338	C	A	0.074	0.162	0.012	3.90E-09	184305	0.001	271.256	-0.023	0.159	0.032	0.480
	rs12202017	G	A	-0.067	0.300	0.010	1.98E-11	184305	0.002	346.224	0.008	0.294	0.026	0.752
	rs1412444	T	C	0.067	0.369	0.010	5.15E-12	184305	0.002	383.968	-0.002	0.345	0.024	0.935
	rs16986953	A	G	0.085	0.105	0.015	1.45E-08	184305	0.001	250.935	0.025	0.068	0.047	0.594
	rs17087335	T	G	0.061	0.215	0.011	4.59E-08	184305	0.001	229.705	-0.011	0.188	0.030	0.721
	rs17678683	G	T	0.099	0.088	0.017	3.00E-09	184305	0.002	288.193	0.025	0.079	0.044	0.562
	rs180803	T	G	-0.181	0.029	0.028	1.64E-10	184305	0.002	343.440	0.163	0.010	0.113	0.149
	rs1870634	G	T	0.076	0.637	0.010	5.55E-15	184305	0.003	491.753	0.029	0.332	0.025	0.245
	rs2107595	A	G	0.073	0.200	0.011	8.05E-11	184305	0.002	318.983	-0.028	0.144	0.032	0.382
	rs2128739	C	A	-0.066	0.676	0.010	7.05E-11	184305	0.002	347.450	0.044	0.274	0.026	0.090
	rs2487928	A	G	0.063	0.418	0.010	4.41E-11	184305	0.002	352.503	0.024	0.446	0.023	0.299
	rs2519093	T	C	0.080	0.191	0.012	1.19E-11	184305	0.002	362.356	-0.038	0.196	0.029	0.195
	rs2681472	G	A	0.074	0.201	0.011	6.17E-11	184305	0.002	326.113	-0.011	0.166	0.032	0.720

	rs28451064	A	G	0.128	0.121	0.016	1.33E-15	184305	0.003	641.097	-0.019	0.131	0.035	0.583
	rs2891168	G	A	0.193	0.489	0.009	2.29E-98	184305	0.019	3510.682	0.011	0.483	0.023	0.638
	rs3918226	T	C	0.133	0.065	0.022	1.69E-09	184305	0.002	396.233	0.039	0.080	0.045	0.382
	rs4420638	G	A	0.092	0.166	0.014	7.07E-11	184305	0.002	432.132	0.020	0.182	0.031	0.526
	rs4468572	C	T	0.077	0.586	0.010	4.44E-16	184305	0.003	535.043	0.010	0.432	0.024	0.666
	rs4593108	G	C	-0.071	0.205	0.012	8.82E-10	184305	0.002	301.494	-0.026	0.173	0.031	0.410
	rs515135	C	T	0.067	0.792	0.012	3.09E-08	184305	0.002	277.090	0.037	0.180	0.030	0.211
	rs55730499	T	C	0.317	0.056	0.024	5.39E-39	184305	0.011	1982.771	0.050	0.079	0.044	0.262
	rs56062135	T	C	-0.070	0.206	0.012	4.52E-09	184305	0.002	293.440	-0.025	0.248	0.027	0.358
	rs56289821	A	G	-0.134	0.100	0.017	4.44E-15	184305	0.003	596.131	0.015	0.122	0.035	0.665
	rs56336142	C	T	-0.067	0.193	0.012	1.85E-08	184305	0.001	256.372	0.024	0.212	0.029	0.406
	rs663129	A	G	0.058	0.257	0.011	3.20E-09	184305	0.001	238.318	0.019	0.236	0.028	0.486
	rs6689306	G	A	-0.056	0.552	0.009	2.60E-09	184305	0.002	286.373	0.011	0.413	0.024	0.658
	rs67180937	G	T	0.079	0.663	0.011	1.01E-12	184305	0.003	512.872	0.032	0.250	0.031	0.305
	rs7212798	C	T	0.080	0.147	0.014	1.88E-08	184305	0.002	295.185	0.047	0.147	0.033	0.157
	rs7528419	G	A	-0.115	0.214	0.011	1.97E-23	184305	0.004	817.381	-0.006	0.213	0.028	0.827
	rs7568458	A	T	0.060	0.449	0.010	3.62E-10	184305	0.002	324.633	-0.054	0.459	0.023	0.021
	rs8042271	A	G	-0.097	0.098	0.018	3.68E-08	184305	0.002	304.472	-0.061	0.037	0.062	0.329
	rs9970807	T	C	-0.126	0.085	0.017	5.00E-14	184305	0.002	453.980	0.006	0.092	0.041	0.891
**CHD (Finn)**	rs1044506	G	T	0.098	0.867	0.016	2.07E-09	218792	0.002	487.409	0.002	0.138	0.033	0.958
	rs10455872	G	A	0.271	0.046	0.027	1.50E-24	218792	0.006	1412.028	0.051	0.077	0.045	0.254
	rs10859837	A	G	-0.099	0.106	0.018	4.14E-08	218792	0.002	407.941	0.008	0.133	0.034	0.819
	rs113113862	A	G	-0.096	0.215	0.014	1.18E-12	218792	0.003	684.909	0.038	0.257	0.027	0.147
	rs11591147	T	G	-0.210	0.036	0.030	2.17E-12	218792	0.003	675.302	0.033	0.018	0.088	0.703
	rs117733303	G	A	0.485	0.011	0.053	9.61E-20	218792	0.005	1157.477	0.059	0.019	0.089	0.510
	rs11852887	C	A	-0.092	0.641	0.012	1.05E-15	218792	0.004	861.125	0.000	0.237	0.028	0.989

	rs137943060	A	G	0.274	0.020	0.041	1.22E-11	218792	0.003	632.901	-0.100	0.021	0.095	0.296
	rs140750546	T	C	0.109	0.102	0.018	2.78E-09	218792	0.002	475.157	0.048	0.129	0.035	0.172
	rs17114046	G	A	-0.100	0.109	0.018	1.79E-08	218792	0.002	425.290	0.009	0.092	0.041	0.820
	rs17477177	C	T	0.084	0.298	0.012	3.35E-12	218792	0.003	646.273	-0.018	0.201	0.029	0.530
	rs2495477	G	A	-0.067	0.436	0.011	2.32E-09	218792	0.002	478.272	-0.013	0.389	0.025	0.592
	rs28451064	A	G	0.113	0.153	0.015	1.79E-13	218792	0.003	729.343	-0.019	0.131	0.035	0.583
	rs3918226	T	C	0.134	0.070	0.022	6.29E-10	218792	0.002	511.257	0.039	0.080	0.045	0.382
	rs4350997	T	C	-0.083	0.785	0.014	8.50E-10	218792	0.002	506.808	-0.020	0.163	0.031	0.525
	rs56225615	T	C	0.085	0.193	0.014	1.21E-09	218792	0.002	493.706	-0.009	0.146	0.033	0.774
	rs653178	T	C	-0.065	0.584	0.011	5.84E-09	218792	0.002	452.853	-0.016	0.593	0.024	0.512
	rs660240	C	T	0.083	0.787	0.014	1.12E-09	218792	0.002	501.252	-0.001	0.206	0.029	0.972
	rs7209564	T	C	0.068	0.718	0.012	3.90E-08	218792	0.002	404.253	0.010	0.279	0.026	0.695
	rs7412	T	C	-0.198	0.054	0.025	1.14E-15	218792	0.004	871.418	-0.077	0.088	0.042	0.064
	rs7668383	C	T	-0.093	0.178	0.015	1.53E-10	218792	0.003	558.754	-0.016	0.184	0.030	0.593
	rs77028772	A	G	0.129	0.128	0.017	6.69E-15	218792	0.004	810.533	0.025	0.141	0.033	0.453
	rs9515203	C	T	-0.077	0.260	0.013	1.13E-09	218792	0.002	496.655	0.007	0.264	0.027	0.807
	rs964184	C	G	-0.095	0.854	0.016	1.20E-09	218792	0.002	490.868	-0.001	0.862	0.035	0.984
	rs9644861	T	C	0.175	0.418	0.011	3.07E-55	218792	0.015	3302.429	0.009	0.494	0.023	0.703
**MI**	rs11591147	T	G	-0.297	0.036	0.043	3.86E-12	200614	0.006	1248.274	0.033	0.018	0.088	0.703
	rs12052201	T	G	-0.120	0.206	0.019	5.79E-10	200614	0.005	942.795	0.034	0.250	0.027	0.210
	rs1537371	A	C	0.214	0.420	0.016	3.41E-42	200614	0.022	4590.376	0.008	0.493	0.023	0.734
	rs17612693	A	T	0.170	0.127	0.024	4.68E-13	200614	0.006	1289.724	0.025	0.142	0.033	0.451
	rs1909196	C	T	0.098	0.711	0.017	1.73E-08	200614	0.004	789.880	0.029	0.317	0.028	0.299
	rs28451064	A	G	0.179	0.153	0.022	2.37E-16	200614	0.008	1670.473	-0.019	0.131	0.035	0.583
	rs3918226	T	C	0.188	0.070	0.031	9.45E-10	200614	0.005	920.052	0.039	0.080	0.045	0.382
	rs4350997	T	C	-0.112	0.786	0.019	3.98E-09	200614	0.004	856.552	-0.020	0.163	0.031	0.525

	rs4887085	C	G	-0.107	0.658	0.017	7.99E-11	200614	0.005	1041.476	-0.002	0.765	0.028	0.935
	rs7412	T	C	-0.229	0.054	0.035	3.33E-11	200614	0.005	1078.162	-0.077	0.088	0.042	0.064
	rs74617384	T	A	0.347	0.045	0.038	4.88E-20	200614	0.010	2109.544	0.090	0.077	0.045	0.044
**Angina pectoris**	rs10455872	G	A	0.347	0.046	0.033	7.26E-26	206008	0.010	2184.817	0.051	0.077	0.045	0.254
	rs10757272	T	C	0.205	0.411	0.014	8.31E-49	206008	0.020	4256.690	0.013	0.481	0.023	0.590
	rs11591147	T	G	-0.249	0.036	0.037	2.37E-11	206008	0.004	898.485	0.033	0.018	0.088	0.703
	rs117733303	G	A	0.467	0.011	0.067	2.49E-12	206008	0.005	994.157	0.059	0.019	0.089	0.510
	rs1261370	A	T	-0.103	0.830	0.018	2.02E-08	206008	0.003	613.507	0.019	0.822	0.031	0.554
	rs12705390	A	G	0.088	0.297	0.015	3.29E-09	206008	0.003	674.970	-0.018	0.201	0.029	0.530
	rs12906835	G	A	-0.115	0.628	0.014	5.26E-16	206008	0.006	1283.804	-0.007	0.236	0.028	0.806
	rs137943060	A	G	0.331	0.019	0.051	5.42E-11	206008	0.004	863.643	-0.100	0.021	0.095	0.296
	rs149714437	A	G	0.121	0.109	0.022	4.95E-08	206008	0.003	589.031	-0.051	0.037	0.063	0.419
	rs56225615	T	C	0.095	0.192	0.017	4.46E-08	206008	0.003	583.374	-0.009	0.146	0.033	0.774
	rs660240	C	T	0.093	0.786	0.017	3.60E-08	206008	0.003	598.348	-0.001	0.206	0.029	0.972
	rs73015021	G	A	-0.165	0.100	0.023	1.21E-12	206008	0.005	1011.201	0.017	0.123	0.035	0.631
	rs7412	T	C	-0.211	0.054	0.031	6.05E-12	206008	0.005	936.072	-0.077	0.088	0.042	0.064
	rs750597	A	T	-0.096	0.321	0.015	6.64E-11	206008	0.004	833.880	-0.005	0.312	0.025	0.838
	rs7608755	A	G	0.121	0.113	0.022	2.56E-08	206008	0.003	608.948	0.054	0.129	0.035	0.120
	rs7668383	C	T	-0.102	0.178	0.018	1.96E-08	206008	0.003	627.743	-0.016	0.184	0.030	0.593
	rs77028772	A	G	0.155	0.127	0.021	4.09E-14	206008	0.005	1107.705	0.025	0.141	0.033	0.453
	rs964184	C	G	-0.108	0.855	0.019	2.36E-08	206008	0.003	603.691	-0.001	0.862	0.035	0.984
	rs9970807	T	C	-0.132	0.109	0.022	2.53E-09	206008	0.003	698.576	0.006	0.092	0.041	0.891
	rs9980618	T	C	0.123	0.136	0.020	9.60E-10	206008	0.004	733.279	-0.002	0.129	0.035	0.960

Supplementary Table S3. Instrumental SNPs of BMI and F statistics.

Instrumental SNP	Effect Allele	Other Allele	EAF	BETA	SE	*P*	Sample size(n)	R^2	F
rs72634826	A	G	0.260	-0.021	0.002	2.10E-19	454884	1.65E-04	74.930
rs4648450	A	C	0.467	-0.015	0.002	1.30E-13	454884	1.09E-04	49.812
rs10799778	G	T	0.834	-0.019	0.003	3.60E-12	454884	9.54E-05	43.401
rs12140153	T	G	0.094	-0.034	0.003	5.70E-22	454884	1.92E-04	87.542
rs116377258	G	A	0.026	0.067	0.006	1.60E-26	454884	2.25E-04	102.291
rs61813324	T	C	0.136	0.029	0.003	6.20E-23	454884	1.97E-04	89.834
rs539515	C	A	0.205	0.050	0.002	7.00E-91	454884	8.06E-04	367.106
rs2076603	A	G	0.644	-0.012	0.002	2.80E-09	454884	6.95E-05	31.609
rs909001	G	C	0.172	0.016	0.003	7.60E-10	454884	7.48E-05	34.030
rs2568958	A	G	0.604	0.022	0.002	1.50E-28	454884	2.42E-04	109.898
rs1890660	A	G	0.725	-0.012	0.002	4.00E-08	454884	5.94E-05	27.015
rs2678204	G	T	0.340	0.024	0.002	9.00E-31	454884	2.63E-04	119.466
rs11122450	G	T	0.612	-0.012	0.002	1.40E-08	454884	6.35E-05	28.894
rs2791643	T	C	0.762	-0.013	0.002	2.80E-08	454884	6.07E-05	27.635
rs6669341	G	A	0.583	-0.017	0.002	9.20E-18	454884	1.45E-04	66.107
rs3845344	T	C	0.391	0.017	0.002	7.40E-17	454884	1.37E-04	62.405
rs11165643	T	C	0.590	0.020	0.002	7.90E-23	454884	1.91E-04	86.704
rs1778830	A	G	0.362	0.014	0.002	8.80E-12	454884	9.23E-05	41.994
rs815163	C	T	0.563	-0.017	0.002	4.60E-17	454884	1.39E-04	63.167
rs4658403	T	C	0.834	-0.019	0.003	2.70E-12	454884	9.66E-05	43.958
rs12072739	G	A	0.225	0.016	0.002	1.20E-11	454884	9.11E-05	41.466
rs7549358	C	G	0.644	-0.011	0.002	3.30E-08	454884	6.02E-05	27.367

rs61828088	A	G	0.110	0.022	0.003	2.90E-12	454884	9.62E-05	43.763
rs76702514	G	C	0.211	-0.017	0.002	1.10E-11	454884	9.23E-05	41.969
rs7539903	A	T	0.615	-0.014	0.002	1.20E-11	454884	9.09E-05	41.351
rs10927006	C	T	0.144	-0.017	0.003	1.60E-09	454884	7.19E-05	32.731
rs3866805	A	C	0.356	0.012	0.002	1.20E-08	454884	6.44E-05	29.278
rs6682438	C	T	0.673	0.013	0.002	1.80E-09	454884	7.12E-05	32.405
rs113603865	T	C	0.212	0.019	0.002	2.50E-14	454884	1.16E-04	52.804
rs1167311	A	G	0.681	-0.019	0.002	1.60E-19	454884	1.63E-04	74.327
rs7519259	A	G	0.528	0.014	0.002	5.80E-12	454884	9.44E-05	42.931
rs34517439	A	C	0.122	0.038	0.003	1.30E-35	454884	3.13E-04	142.578
rs6688826	C	T	0.298	0.014	0.002	7.70E-11	454884	8.33E-05	37.880
rs12089815	A	G	0.549	-0.013	0.002	1.90E-10	454884	8.04E-05	36.570
rs12033257	G	A	0.382	-0.016	0.002	5.10E-14	454884	1.14E-04	51.639
rs75035127	G	A	0.030	-0.041	0.006	1.20E-12	454884	9.96E-05	45.332
rs7516554	T	C	0.400	0.012	0.002	1.50E-09	454884	7.22E-05	32.847
rs62107261	C	T	0.048	-0.092	0.005	1.70E-86	454884	7.70E-04	350.746
rs935166	A	G	0.507	-0.016	0.002	5.90E-16	454884	1.29E-04	58.830
rs6713781	C	G	0.402	-0.013	0.002	1.40E-10	454884	8.27E-05	37.611
rs4671328	G	T	0.551	-0.021	0.002	1.80E-25	454884	2.18E-04	99.097
rs4672338	T	C	0.336	0.013	0.002	2.40E-10	454884	7.91E-05	35.998
rs6752979	A	G	0.317	0.012	0.002	1.10E-08	454884	6.42E-05	29.194
rs10182416	G	A	0.512	0.013	0.002	1.80E-11	454884	8.90E-05	40.478
rs1446585	G	A	0.245	-0.014	0.002	9.70E-10	454884	7.08E-05	32.219
rs62176993	A	G	0.401	0.011	0.002	3.40E-08	454884	6.06E-05	27.553
rs62190049	C	G	0.390	-0.011	0.002	3.70E-08	454884	6.07E-05	27.604
rs1064213	A	G	0.478	0.015	0.002	6.60E-14	454884	1.11E-04	50.336

rs4482463	A	C	0.923	-0.031	0.004	1.40E-16	454884	1.35E-04	61.610
rs6710091	G	C	0.348	-0.012	0.002	2.70E-08	454884	6.08E-05	27.652
rs7571496	G	A	0.261	-0.015	0.002	1.10E-11	454884	9.19E-05	41.804
rs10169594	C	T	0.363	0.012	0.002	1.80E-08	454884	6.28E-05	28.553
rs6707827	G	A	0.704	0.012	0.002	1.70E-08	454884	6.35E-05	28.876
rs11691869	A	C	0.362	-0.020	0.002	1.90E-21	454884	1.79E-04	81.568
rs2381404	C	T	0.244	0.014	0.002	5.50E-09	454884	6.73E-05	30.630
rs6430068	A	G	0.109	0.019	0.003	5.60E-09	454884	6.79E-05	30.873
rs429343	G	A	0.577	-0.017	0.002	8.40E-18	454884	1.46E-04	66.440
rs62176243	T	A	0.245	-0.015	0.002	6.20E-11	454884	8.41E-05	38.257
rs56133507	G	T	0.197	0.014	0.002	1.40E-08	454884	6.34E-05	28.824
rs34234296	A	G	0.392	-0.015	0.002	2.80E-13	454884	1.07E-04	48.862
rs55658481	A	G	0.339	0.013	0.002	1.80E-10	454884	8.00E-05	36.391
rs6744646	G	A	0.828	0.055	0.003	1.70E-98	454884	8.74E-04	397.877
rs59086897	A	T	0.488	0.033	0.002	6.60E-64	454884	5.61E-04	255.160
rs2861685	C	T	0.412	-0.017	0.002	4.10E-17	454884	1.39E-04	63.152
rs12692596	T	C	0.372	0.014	0.002	1.90E-11	454884	8.88E-05	40.379
rs2216931	A	C	0.662	0.017	0.002	2.40E-15	454884	1.24E-04	56.422
rs13427822	G	A	0.271	-0.018	0.002	4.70E-16	454884	1.33E-04	60.477
rs13012070	A	G	0.228	-0.014	0.002	1.80E-09	454884	7.13E-05	32.428
rs35809007	A	G	0.363	-0.017	0.002	4.10E-17	454884	1.40E-04	63.882
rs6545714	A	G	0.601	-0.021	0.002	2.50E-24	454884	2.05E-04	93.214
rs4832298	T	C	0.686	-0.016	0.002	8.20E-14	454884	1.10E-04	49.970
rs11675464	G	A	0.563	0.012	0.002	2.80E-09	454884	6.96E-05	31.651
rs10172070	T	C	0.146	0.016	0.003	9.60E-09	454884	6.47E-05	29.449
rs16846140	G	A	0.338	0.014	0.002	6.10E-11	454884	8.47E-05	38.528

rs13420048	A	C	0.365	-0.016	0.002	3.10E-14	454884	1.14E-04	52.031
rs6705567	C	T	0.376	-0.014	0.002	2.40E-12	454884	9.82E-05	44.666
rs745249	T	C	0.282	0.018	0.002	3.30E-16	454884	1.32E-04	60.133
rs72617140	C	A	0.214	0.019	0.002	1.10E-14	454884	1.18E-04	53.570
rs2433733	A	G	0.678	-0.018	0.002	1.00E-16	454884	1.36E-04	61.849
rs4625852	A	G	0.205	-0.014	0.002	2.00E-08	454884	6.23E-05	28.336
rs62246311	A	G	0.102	0.021	0.003	2.90E-10	454884	7.85E-05	35.718
rs13076052	G	C	0.275	0.014	0.002	3.10E-10	454884	7.93E-05	36.078
rs56038322	A	G	0.311	0.014	0.002	6.80E-11	454884	8.54E-05	38.871
rs11708540	A	G	0.157	0.016	0.003	4.70E-09	454884	6.86E-05	31.225
rs11914525	G	A	0.354	-0.018	0.002	3.70E-18	454884	1.49E-04	67.924
rs1436348	G	A	0.583	0.016	0.002	6.50E-15	454884	1.20E-04	54.651
rs76183894	C	T	0.081	-0.022	0.004	2.70E-09	454884	7.08E-05	32.215
rs13317303	A	C	0.150	-0.016	0.003	1.00E-08	454884	6.51E-05	29.593
rs4017425	T	C	0.470	-0.013	0.002	1.60E-10	454884	8.10E-05	36.867
rs9843653	C	T	0.512	0.029	0.002	7.70E-49	454884	4.27E-04	194.258
rs73052033	C	T	0.185	-0.030	0.003	1.00E-31	454884	2.72E-04	123.923
rs2920503	T	C	0.285	-0.014	0.002	1.40E-10	454884	8.22E-05	37.385
rs9860326	G	C	0.328	0.015	0.002	7.50E-12	454884	9.29E-05	42.245
rs6419869	G	T	0.886	0.022	0.003	2.00E-12	454884	9.76E-05	44.413
rs11709402	G	A	0.279	0.023	0.002	9.50E-25	454884	2.10E-04	95.535
rs1471740	C	T	0.740	0.019	0.002	9.90E-18	454884	1.46E-04	66.315
rs2035936	T	G	0.056	0.036	0.004	9.40E-17	454884	1.41E-04	63.933
rs529200	G	A	0.528	0.017	0.002	5.80E-18	454884	1.48E-04	67.190
rs11919665	T	A	0.680	-0.013	0.002	1.10E-09	454884	7.34E-05	33.391
rs6777784	T	G	0.617	0.012	0.002	1.30E-09	454884	7.25E-05	32.981

rs75557510	G	A	0.061	-0.031	0.004	1.60E-13	454884	1.10E-04	50.207
rs1454687	G	C	0.515	-0.021	0.002	5.50E-26	454884	2.20E-04	99.872
rs1471093	A	G	0.617	0.013	0.002	5.40E-11	454884	8.59E-05	39.095
rs2569993	C	T	0.320	0.013	0.002	1.20E-09	454884	7.36E-05	33.483
rs62241847	G	A	0.315	-0.012	0.002	5.80E-09	454884	6.72E-05	30.576
rs7619139	A	T	0.589	0.013	0.002	1.30E-10	454884	8.21E-05	37.365
rs754635	G	C	0.887	0.023	0.003	5.20E-13	454884	1.03E-04	46.804
rs9852062	A	T	0.557	-0.014	0.002	3.60E-12	454884	9.62E-05	43.784
rs17639546	A	G	0.148	-0.023	0.003	6.70E-17	454884	1.38E-04	62.813
rs9835772	T	A	0.244	0.017	0.002	6.80E-13	454884	1.02E-04	46.394
rs3851998	G	C	0.743	-0.014	0.002	1.20E-09	454884	7.37E-05	33.547
rs355777	C	G	0.408	0.016	0.002	2.10E-14	454884	1.16E-04	52.819
rs6444950	A	G	0.238	0.016	0.002	3.90E-12	454884	9.53E-05	43.368
rs2606228	C	A	0.646	-0.013	0.002	1.50E-10	454884	8.28E-05	37.647
rs6774894	A	T	0.358	0.013	0.002	4.30E-10	454884	7.70E-05	35.016
rs34811474	A	G	0.231	-0.028	0.002	3.30E-33	454884	2.85E-04	129.738
rs4261944	G	T	0.365	0.014	0.002	7.90E-12	454884	9.31E-05	42.359
rs6531639	A	G	0.248	-0.014	0.002	3.00E-09	454884	7.29E-05	33.147
rs2164300	T	C	0.519	-0.012	0.002	8.60E-10	454884	7.49E-05	34.070
rs113079574	T	C	0.193	-0.016	0.003	2.20E-10	454884	8.06E-05	36.646
rs4148155	G	A	0.113	-0.022	0.003	7.80E-13	454884	1.01E-04	45.956
rs17289010	G	A	0.328	-0.014	0.002	1.70E-10	454884	8.07E-05	36.704
rs2051559	C	T	0.133	0.021	0.003	2.70E-12	454884	9.73E-05	44.242
rs10938398	A	G	0.433	0.029	0.002	4.00E-48	454884	4.22E-04	192.170
rs2192158	G	A	0.553	-0.015	0.002	1.40E-14	454884	1.17E-04	53.116
rs1346841	A	G	0.405	-0.013	0.002	2.90E-10	454884	7.91E-05	35.971

rs4419475	T	A	0.407	0.012	0.002	4.90E-09	454884	6.78E-05	30.860
rs1229984	C	T	0.973	0.039	0.006	1.10E-10	454884	8.03E-05	36.512
rs13107325	T	C	0.075	0.047	0.004	5.40E-36	454884	3.11E-04	141.617
rs1296328	C	A	0.559	-0.018	0.002	2.10E-19	454884	1.62E-04	73.851
rs73213484	T	A	0.141	-0.023	0.003	1.00E-15	454884	1.28E-04	58.071
rs2102278	G	A	0.323	0.012	0.002	2.70E-08	454884	6.12E-05	27.841
rs11099020	T	C	0.641	-0.014	0.002	4.90E-12	454884	9.49E-05	43.168
rs66679256	T	C	0.446	0.015	0.002	1.90E-14	454884	1.16E-04	52.829
rs7442137	T	C	0.634	-0.012	0.002	1.60E-09	454884	7.22E-05	32.866
rs9991259	A	G	0.633	0.011	0.002	4.40E-08	454884	5.94E-05	27.026
rs6843852	T	C	0.508	0.013	0.002	1.70E-11	454884	8.98E-05	40.840
rs55920177	T	A	0.129	-0.018	0.003	8.70E-10	454884	7.46E-05	33.959
rs698147	G	A	0.543	-0.013	0.002	2.90E-11	454884	8.78E-05	39.926
rs111689389	C	G	0.283	-0.014	0.002	7.40E-10	454884	7.52E-05	34.204
rs1919243	C	T	0.488	0.012	0.002	7.10E-09	454884	6.81E-05	30.989
rs1582931	A	G	0.473	-0.013	0.002	1.60E-10	454884	8.24E-05	37.466
rs1438945	A	T	0.715	-0.014	0.002	4.90E-10	454884	7.74E-05	35.190
rs13176429	C	T	0.688	0.015	0.002	6.10E-12	454884	9.37E-05	42.606
rs116374395	A	G	0.035	0.032	0.005	3.60E-09	454884	6.95E-05	31.605
rs396755	G	C	0.571	-0.012	0.002	6.90E-10	454884	7.56E-05	34.399
rs10063055	T	C	0.253	0.013	0.002	1.40E-08	454884	6.37E-05	28.971
rs1503526	C	T	0.480	0.016	0.002	5.30E-15	454884	1.21E-04	55.087
rs1459190	A	G	0.519	-0.014	0.002	6.30E-13	454884	1.04E-04	47.519
rs252761	T	G	0.588	-0.011	0.002	2.70E-08	454884	6.18E-05	28.126
rs2962334	T	G	0.020	0.043	0.007	8.40E-10	454884	7.44E-05	33.840
rs1477290	C	T	0.137	0.034	0.003	4.20E-31	454884	2.71E-04	123.342

rs159037	C	T	0.254	0.013	0.002	2.60E-08	454884	6.13E-05	27.888
rs347551	G	C	0.472	0.014	0.002	8.40E-12	454884	9.54E-05	43.415
rs11134679	G	A	0.685	0.019	0.002	5.50E-18	454884	1.49E-04	67.733
rs2307111	C	T	0.395	-0.028	0.002	1.80E-42	454884	3.70E-04	168.364
rs40071	C	T	0.179	-0.026	0.003	1.30E-23	454884	2.00E-04	90.915
rs17056301	C	T	0.256	0.013	0.002	8.00E-09	454884	6.63E-05	30.180
rs7707394	A	G	0.357	-0.019	0.002	1.10E-20	454884	1.72E-04	78.127
rs28404639	T	C	0.366	-0.012	0.002	5.80E-09	454884	6.74E-05	30.670
rs7442885	G	C	0.214	-0.023	0.002	5.20E-21	454884	1.76E-04	79.978
rs62379271	G	T	0.578	0.012	0.002	7.30E-09	454884	6.66E-05	30.304
rs7704382	G	C	0.433	0.012	0.002	1.30E-09	454884	7.30E-05	33.225
rs329118	T	C	0.419	-0.017	0.002	1.00E-16	454884	1.37E-04	62.229
rs2133561	T	A	0.611	-0.014	0.002	5.90E-12	454884	9.58E-05	43.582
rs4958702	C	T	0.572	-0.016	0.002	9.60E-15	454884	1.19E-04	53.974
rs1322842	G	A	0.609	-0.013	0.002	1.10E-10	454884	8.35E-05	37.968
rs75499503	T	C	0.220	-0.018	0.002	3.10E-13	454884	1.08E-04	49.298
rs9366863	C	T	0.672	-0.028	0.002	2.60E-40	454884	3.49E-04	158.786
rs1266874	G	A	0.350	0.014	0.002	1.20E-11	454884	9.10E-05	41.404
rs6938973	C	T	0.602	0.018	0.002	1.90E-19	454884	1.61E-04	73.446
rs6922607	G	A	0.190	0.014	0.003	1.50E-08	454884	6.33E-05	28.795
rs6909685	T	C	0.327	-0.015	0.002	6.20E-12	454884	9.43E-05	42.920
rs156201	C	G	0.753	0.013	0.002	2.50E-08	454884	6.14E-05	27.924
rs13218383	G	C	0.335	-0.015	0.002	7.40E-13	454884	1.02E-04	46.352
rs7762794	G	A	0.286	0.015	0.002	2.00E-11	454884	8.89E-05	40.460
rs2178899	T	A	0.129	-0.026	0.003	4.80E-18	454884	1.48E-04	67.413
rs2281819	A	T	0.230	-0.016	0.002	3.60E-11	454884	8.71E-05	39.602

rs34045288	T	C	0.334	0.024	0.002	6.90E-29	454884	2.46E-04	112.144
rs9296389	C	G	0.411	0.012	0.002	1.10E-08	454884	6.44E-05	29.295
rs1327259	G	A	0.388	-0.015	0.002	1.50E-13	454884	1.09E-04	49.474
rs57989773	C	T	0.245	0.013	0.002	1.60E-08	454884	6.70E-05	30.473
rs2253310	G	C	0.626	0.018	0.002	2.40E-18	454884	1.51E-04	68.770
rs2875762	C	G	0.243	0.015	0.002	9.60E-11	454884	8.36E-05	38.045
rs765874	A	T	0.489	-0.012	0.002	3.60E-09	454884	6.90E-05	31.385
rs11757278	C	T	0.304	-0.015	0.002	1.00E-11	454884	9.17E-05	41.713
rs9267671	A	G	0.061	0.026	0.004	5.30E-10	454884	7.64E-05	34.753
rs72892910	T	G	0.172	0.039	0.003	6.60E-49	454884	4.29E-04	195.345
rs72948836	G	A	0.059	-0.024	0.004	7.00E-09	454884	6.64E-05	30.208
rs9294260	A	G	0.477	0.015	0.002	2.00E-13	454884	1.08E-04	49.136
rs4895799	T	C	0.585	0.012	0.002	1.10E-08	454884	6.50E-05	29.582
rs2781668	T	C	0.166	0.015	0.003	3.20E-08	454884	6.07E-05	27.594
rs72910629	G	A	0.136	0.016	0.003	2.50E-08	454884	6.22E-05	28.276
rs9478496	C	T	0.164	0.018	0.003	6.60E-11	454884	8.50E-05	38.668
rs36007635	A	G	0.138	-0.021	0.003	1.20E-12	454884	1.00E-04	45.494
rs3807566	T	G	0.438	-0.013	0.002	4.30E-10	454884	7.76E-05	35.301
rs34025316	T	C	0.338	0.012	0.002	2.60E-08	454884	6.21E-05	28.271
rs2190887	T	C	0.561	-0.011	0.002	4.10E-08	454884	5.99E-05	27.227
rs58862095	T	C	0.419	-0.023	0.002	4.90E-29	454884	2.49E-04	113.413
rs17149254	C	T	0.805	-0.021	0.003	3.90E-16	454884	1.38E-04	62.953
rs7805441	T	C	0.502	0.013	0.002	2.60E-11	454884	8.93E-05	40.628
rs3901286	A	C	0.152	-0.023	0.003	3.70E-16	454884	1.32E-04	60.170
rs2396625	A	T	0.421	-0.020	0.002	4.40E-22	454884	1.87E-04	85.197
rs11525873	C	T	0.098	-0.024	0.003	8.50E-13	454884	1.02E-04	46.364

rs16868443	C	G	0.360	0.012	0.002	7.90E-09	454884	6.61E-05	30.058
rs2103123	T	A	0.472	0.011	0.002	4.30E-08	454884	6.14E-05	27.949
rs6950388	A	G	0.795	0.016	0.002	9.20E-11	454884	8.32E-05	37.870
rs17132130	C	G	0.222	-0.018	0.002	2.70E-13	454884	1.06E-04	48.417
rs4722398	T	C	0.136	0.019	0.003	1.40E-10	454884	8.13E-05	37.000
rs9638713	G	A	0.975	-0.036	0.006	2.40E-08	454884	6.28E-05	28.568
rs4307239	G	A	0.459	0.012	0.002	4.60E-10	454884	7.73E-05	35.178
rs213518	C	T	0.146	0.016	0.003	8.40E-09	454884	6.59E-05	29.984
rs215634	G	A	0.612	-0.015	0.002	4.10E-14	454884	1.14E-04	51.873
rs4718964	T	G	0.413	0.011	0.002	2.60E-08	454884	6.19E-05	28.145
rs10950301	A	G	0.182	-0.016	0.003	3.80E-10	454884	7.77E-05	35.330
rs6962980	C	A	0.556	-0.016	0.002	1.60E-15	454884	1.26E-04	57.256
rs2289379	T	C	0.396	-0.015	0.002	1.80E-13	454884	1.08E-04	49.269
rs1805123	G	T	0.245	-0.016	0.002	1.30E-12	454884	9.98E-05	45.403
rs13248187	C	T	0.268	0.016	0.002	1.50E-12	454884	1.00E-04	45.652
rs4737188	T	A	0.474	-0.013	0.002	7.40E-11	454884	8.44E-05	38.394
rs72673947	G	A	0.107	0.022	0.003	7.70E-12	454884	9.39E-05	42.728
rs56893062	G	T	0.303	0.013	0.002	2.40E-09	454884	7.08E-05	32.220
rs36061954	T	C	0.399	0.013	0.002	2.50E-10	454884	7.95E-05	36.160
rs35957544	T	G	0.574	-0.020	0.002	1.90E-22	454884	1.89E-04	86.177
rs1609010	G	A	0.566	0.022	0.002	7.10E-27	454884	2.29E-04	104.195
rs1106761	A	G	0.384	0.015	0.002	1.70E-12	454884	1.01E-04	46.101
rs2616143	A	G	0.320	-0.014	0.002	1.60E-10	454884	8.17E-05	37.180
rs12156160	G	A	0.150	0.016	0.003	1.60E-08	454884	6.37E-05	28.962
rs2922757	T	A	0.597	0.013	0.002	4.50E-10	454884	7.74E-05	35.226
rs4876611	G	A	0.720	0.019	0.002	3.50E-18	454884	1.50E-04	68.347

rs10099330	G	A	0.453	0.012	0.002	3.70E-09	454884	6.91E-05	31.456
rs11250094	C	G	0.548	-0.021	0.002	4.10E-25	454884	2.14E-04	97.296
rs2725371	G	A	0.696	-0.016	0.002	3.50E-13	454884	1.06E-04	48.122
rs12681792	A	C	0.193	0.015	0.003	4.40E-09	454884	6.88E-05	31.316
rs76387394	G	A	0.053	0.027	0.004	1.00E-09	454884	7.42E-05	33.758
rs2114210	A	G	0.336	0.014	0.002	7.10E-11	454884	8.46E-05	38.485
rs7828631	T	C	0.110	0.018	0.003	1.20E-08	454884	6.47E-05	29.417
rs7042372	G	A	0.335	-0.013	0.002	2.70E-09	454884	7.09E-05	32.232
rs10756792	T	C	0.743	-0.019	0.002	2.40E-17	454884	1.44E-04	65.357
rs16916303	G	A	0.120	-0.020	0.003	3.10E-10	454884	8.04E-05	36.559
rs1360201	T	C	0.482	0.013	0.002	4.00E-11	454884	8.65E-05	39.334
rs2482356	C	T	0.429	-0.011	0.002	2.80E-08	454884	6.10E-05	27.754
rs10760277	T	C	0.385	0.014	0.002	7.90E-12	454884	9.34E-05	42.511
rs7027304	T	C	0.653	0.015	0.002	1.70E-12	454884	9.97E-05	45.356
rs56203622	C	T	0.146	0.018	0.003	2.00E-10	454884	8.02E-05	36.473
rs12375985	A	G	0.355	-0.015	0.002	1.50E-12	454884	9.94E-05	45.226
rs13292699	C	A	0.434	-0.021	0.002	4.00E-26	454884	2.23E-04	101.283
rs12001437	C	T	0.368	0.012	0.002	2.20E-09	454884	7.10E-05	32.300
rs7852189	G	A	0.316	0.017	0.002	1.10E-15	454884	1.27E-04	57.839
rs1412239	G	C	0.323	0.025	0.002	6.40E-31	454884	2.65E-04	120.375
rs1999433	T	C	0.447	-0.012	0.002	3.70E-09	454884	6.93E-05	31.526
rs7038943	C	T	0.339	-0.014	0.002	1.50E-11	454884	9.02E-05	41.045
rs28670671	C	T	0.286	-0.013	0.002	4.00E-08	454884	6.41E-05	29.139
rs1330199	T	G	0.483	-0.012	0.002	2.80E-09	454884	7.07E-05	32.154
rs7034554	G	A	0.374	-0.014	0.002	4.70E-11	454884	8.58E-05	39.027
rs7357754	G	A	0.500	0.014	0.002	5.80E-13	454884	1.04E-04	47.155

rs2398861	G	A	0.259	0.018	0.002	1.60E-15	454884	1.27E-04	57.887
rs7024334	G	T	0.779	-0.014	0.002	1.70E-08	454884	6.31E-05	28.699
rs12762034	C	T	0.077	0.028	0.004	1.30E-13	454884	1.09E-04	49.513
rs10510025	T	C	0.247	0.018	0.002	5.00E-15	454884	1.22E-04	55.620
rs117118217	C	G	0.018	0.044	0.008	2.30E-08	454884	6.85E-05	31.178
rs2172131	C	T	0.579	-0.015	0.002	6.70E-14	454884	1.12E-04	50.779
rs10903791	A	G	0.604	0.011	0.002	4.50E-08	454884	5.97E-05	27.154
rs11012732	G	A	0.332	0.021	0.002	3.30E-24	454884	2.05E-04	93.115
rs4919197	T	C	0.474	0.011	0.002	4.80E-08	454884	6.05E-05	27.524
rs7893571	T	G	0.666	0.014	0.002	9.40E-12	454884	9.27E-05	42.149
rs7070670	T	C	0.328	-0.013	0.002	2.70E-09	454884	7.12E-05	32.370
rs7924036	T	G	0.503	-0.014	0.002	3.50E-13	454884	1.05E-04	47.753
rs11000993	C	T	0.124	0.021	0.003	1.80E-12	454884	9.83E-05	44.709
rs577525	C	T	0.562	0.019	0.002	3.70E-22	454884	1.86E-04	84.585
rs7916385	T	C	0.150	-0.024	0.003	1.80E-15	454884	1.48E-04	67.519
rs12259464	A	G	0.485	0.013	0.002	5.70E-11	454884	8.59E-05	39.083
rs147568678	C	T	0.238	-0.014	0.002	1.40E-09	454884	7.32E-05	33.310
rs11017771	C	G	0.206	-0.014	0.002	2.00E-08	454884	6.28E-05	28.572
rs73601548	T	C	0.115	0.017	0.003	4.90E-08	454884	5.95E-05	27.079
rs35972789	A	C	0.037	-0.029	0.005	2.90E-08	454884	6.09E-05	27.718
rs17399739	G	A	0.069	0.028	0.004	2.00E-12	454884	9.85E-05	44.825
rs10887578	C	G	0.498	0.013	0.002	8.40E-11	454884	8.47E-05	38.509
rs79780963	T	C	0.077	0.024	0.004	2.50E-10	454884	7.92E-05	36.050
rs705145	A	C	0.345	0.014	0.002	1.60E-11	454884	9.01E-05	40.990
rs67609008	C	T	0.284	0.018	0.002	2.80E-15	454884	1.25E-04	56.762
rs4757144	A	G	0.590	0.016	0.002	1.50E-15	454884	1.26E-04	57.454

rs11024271	C	T	0.623	0.012	0.002	2.00E-08	454884	6.25E-05	28.452
rs6265	T	C	0.188	-0.040	0.003	5.90E-56	454884	4.92E-04	224.064
rs594024	C	T	0.554	-0.015	0.002	1.00E-13	454884	1.10E-04	50.056
rs7944782	G	T	0.510	0.016	0.002	1.40E-15	454884	1.28E-04	58.082
rs558887	G	A	0.308	-0.013	0.002	8.00E-10	454884	7.54E-05	34.283
rs61903695	G	A	0.255	0.017	0.002	2.70E-13	454884	1.06E-04	48.362
rs1793636	C	G	0.309	-0.013	0.002	6.00E-10	454884	7.62E-05	34.643
rs7952102	C	T	0.388	-0.014	0.002	2.80E-12	454884	9.66E-05	43.923
rs4929923	C	T	0.645	0.019	0.002	3.40E-20	454884	1.68E-04	76.342
rs13642	T	A	0.361	-0.016	0.002	1.80E-15	454884	1.25E-04	56.979
rs59227842	G	A	0.311	0.023	0.002	1.90E-26	454884	2.28E-04	103.948
rs55707359	G	T	0.015	0.053	0.008	1.10E-10	454884	8.49E-05	38.632
rs7124681	A	C	0.408	0.026	0.002	1.40E-37	454884	3.24E-04	147.476
rs2234458	T	C	0.640	-0.021	0.002	1.10E-23	454884	1.99E-04	90.659
rs10160769	C	G	0.217	-0.015	0.002	4.00E-10	454884	7.93E-05	36.054
rs2512892	C	T	0.566	0.013	0.002	5.50E-11	454884	8.56E-05	38.939
rs12364470	G	T	0.165	0.019	0.003	1.10E-12	454884	1.00E-04	45.717
rs10742752	C	T	0.612	0.012	0.002	1.00E-08	454884	6.50E-05	29.585
rs7947143	A	G	0.163	-0.018	0.003	3.60E-11	454884	8.70E-05	39.573
rs1048932	A	C	0.413	-0.015	0.002	2.10E-14	454884	1.16E-04	52.771
rs7925100	A	G	0.396	0.014	0.002	1.10E-12	454884	1.00E-04	45.681
rs349071	A	G	0.500	-0.013	0.002	9.20E-11	454884	8.37E-05	38.098
rs329651	T	G	0.804	0.016	0.003	4.80E-10	454884	7.76E-05	35.312
rs55726687	A	G	0.210	0.025	0.002	2.70E-24	454884	2.05E-04	93.184
rs6560906	C	T	0.692	-0.012	0.002	1.70E-08	454884	6.30E-05	28.677
rs1458156	T	C	0.488	0.014	0.002	3.10E-12	454884	9.67E-05	43.987

rs1126930	C	G	0.035	0.032	0.005	4.60E-09	454884	6.81E-05	30.988
rs3897102	T	C	0.411	0.012	0.002	1.90E-09	454884	7.29E-05	33.165
rs7132908	A	G	0.384	0.029	0.002	1.10E-46	454884	4.09E-04	186.180
rs4267103	C	T	0.186	0.016	0.003	5.40E-10	454884	7.67E-05	34.909
rs317656	A	T	0.725	-0.014	0.002	1.20E-10	454884	8.24E-05	37.497
rs55966114	T	C	0.193	0.015	0.003	3.80E-09	454884	6.93E-05	31.528
rs4764949	G	A	0.326	-0.018	0.002	3.10E-17	454884	1.42E-04	64.536
rs961498	C	G	0.503	0.012	0.002	2.20E-09	454884	7.22E-05	32.835
rs147730268	T	G	0.087	-0.035	0.004	9.80E-22	454884	1.91E-04	86.685
rs10505836	C	A	0.860	0.018	0.003	7.60E-10	454884	7.62E-05	34.687
rs78086698	C	T	0.040	0.032	0.005	2.30E-10	454884	8.04E-05	36.567
rs723672	T	C	0.432	0.011	0.002	4.00E-08	454884	6.05E-05	27.537
rs2271189	A	G	0.403	-0.016	0.002	2.60E-15	454884	1.25E-04	56.680
rs11115160	A	G	0.238	-0.013	0.002	2.40E-08	454884	6.25E-05	28.452
rs12427047	T	C	0.243	-0.017	0.002	1.90E-13	454884	1.07E-04	48.860
rs73193736	G	A	0.244	-0.018	0.002	3.60E-15	454884	1.25E-04	56.779
rs11610621	A	T	0.148	0.016	0.003	5.00E-09	454884	6.79E-05	30.868
rs56858768	A	G	0.297	0.016	0.002	5.60E-13	454884	1.04E-04	47.364
rs11842871	T	G	0.260	-0.015	0.002	2.00E-11	454884	8.97E-05	40.787
rs4477562	T	C	0.129	0.030	0.003	3.70E-23	454884	1.99E-04	90.331
rs9888533	T	C	0.538	0.012	0.002	2.20E-09	454884	7.35E-05	33.450
rs1183668	G	C	0.370	-0.016	0.002	1.90E-14	454884	1.18E-04	53.501
rs1967772	A	G	0.285	-0.017	0.002	3.10E-14	454884	1.16E-04	52.723
rs17446299	G	C	0.166	0.016	0.003	1.90E-09	454884	7.18E-05	32.649
rs1441264	A	G	0.594	0.018	0.002	3.70E-18	454884	1.57E-04	71.264
rs7996639	A	G	0.449	0.014	0.002	1.20E-12	454884	1.02E-04	46.323

rs56399737	T	C	0.449	-0.016	0.002	3.60E-15	454884	1.24E-04	56.391
rs6561937	A	T	0.754	-0.016	0.002	6.60E-12	454884	9.43E-05	42.882
rs4055791	T	C	0.417	-0.018	0.002	4.40E-19	454884	1.59E-04	72.165
rs7331420	A	G	0.285	-0.014	0.002	1.60E-10	454884	8.19E-05	37.258
rs9571687	A	C	0.329	-0.013	0.002	4.60E-10	454884	7.75E-05	35.273
rs9522173	T	A	0.394	-0.012	0.002	1.30E-09	454884	7.31E-05	33.249
rs8015400	A	C	0.677	0.021	0.002	9.60E-24	454884	2.01E-04	91.326
rs1451963	T	G	0.082	0.023	0.004	3.10E-10	454884	7.89E-05	35.912
rs1286058	A	T	0.704	0.015	0.002	1.70E-11	454884	9.02E-05	41.055
rs10144067	T	C	0.591	0.018	0.002	1.50E-19	454884	1.65E-04	75.138
rs12881629	G	A	0.083	0.022	0.004	8.30E-10	454884	7.47E-05	33.999
rs12889639	A	G	0.651	0.016	0.002	5.80E-14	454884	1.12E-04	51.115
rs12885458	G	T	0.508	-0.016	0.002	1.80E-16	454884	1.35E-04	61.504
rs3902951	G	T	0.237	0.015	0.002	8.80E-10	454884	7.64E-05	34.753
rs1860750	A	T	0.511	0.012	0.002	4.00E-09	454884	6.90E-05	31.386
rs8020365	A	T	0.220	0.025	0.002	6.70E-25	454884	2.13E-04	96.733
rs6575340	A	G	0.636	0.021	0.002	9.10E-24	454884	2.02E-04	91.829
rs3803286	G	A	0.667	-0.018	0.002	3.10E-18	454884	1.51E-04	68.547
rs113624107	A	G	0.226	0.014	0.002	1.50E-09	454884	7.28E-05	33.112
rs7159965	G	C	0.164	0.015	0.003	3.60E-08	454884	6.06E-05	27.581
rs217672	C	A	0.272	0.017	0.002	2.90E-14	454884	1.16E-04	52.549
rs61992671	G	A	0.492	-0.016	0.002	1.20E-14	454884	1.29E-04	58.792
rs62007782	A	G	0.265	-0.016	0.002	6.80E-13	454884	1.03E-04	46.677
rs28457680	A	T	0.137	0.018	0.003	6.60E-10	454884	7.67E-05	34.879
rs3784710	C	T	0.227	-0.030	0.002	2.30E-35	454884	3.05E-04	138.925
rs35364449	T	C	0.110	0.022	0.003	6.70E-12	454884	9.47E-05	43.077

rs7169847	T	G	0.636	-0.014	0.002	2.00E-11	454884	9.01E-05	40.990
rs4284600	C	T	0.467	0.012	0.002	1.30E-09	454884	7.41E-05	33.706
rs34153025	C	T	0.022	-0.038	0.007	1.80E-08	454884	6.41E-05	29.166
rs79212998	G	T	0.069	-0.023	0.004	2.80E-09	454884	7.06E-05	32.118
rs8024137	T	A	0.848	0.016	0.003	2.40E-08	454884	6.23E-05	28.363
rs9944241	C	T	0.484	-0.015	0.002	2.50E-12	454884	1.06E-04	48.184
rs11639144	A	G	0.238	-0.014	0.002	8.10E-09	454884	6.66E-05	30.309
rs140159717	T	C	0.082	-0.025	0.004	3.90E-11	454884	9.21E-05	41.906
rs7498044	A	G	0.217	-0.017	0.002	4.70E-12	454884	9.71E-05	44.157
rs355393	G	A	0.470	-0.012	0.002	8.50E-09	454884	6.61E-05	30.083
rs11071646	A	G	0.022	-0.040	0.007	4.80E-09	454884	6.85E-05	31.141
rs57488047	C	T	0.468	-0.015	0.002	4.50E-14	454884	1.15E-04	52.288
rs56803094	G	A	0.227	-0.015	0.002	5.00E-10	454884	7.75E-05	35.249
rs3211995	A	G	0.160	-0.015	0.003	1.70E-08	454884	6.36E-05	28.954
rs4613074	C	T	0.185	-0.024	0.003	3.70E-20	454884	1.68E-04	76.344
rs35154326	G	A	0.274	-0.013	0.002	2.80E-09	454884	7.12E-05	32.380
rs56094641	G	A	0.405	0.073	0.002	1.00E-200	454884	2.59E-03	1182.038
rs9673839	G	A	0.491	0.013	0.002	8.80E-11	454884	8.44E-05	38.380
rs7201895	A	G	0.354	-0.015	0.002	8.00E-13	454884	1.03E-04	46.876
rs249293	C	G	0.696	0.013	0.002	2.10E-09	454884	7.19E-05	32.706
rs5011579	G	C	0.715	0.014	0.002	1.30E-10	454884	8.20E-05	37.323
rs12149660	A	G	0.115	-0.022	0.003	1.60E-12	454884	1.00E-04	45.596
rs7206608	G	C	0.322	0.013	0.002	2.60E-10	454884	7.93E-05	36.094
rs3814883	T	C	0.482	0.024	0.002	2.50E-33	454884	2.89E-04	131.439
rs2342892	G	T	0.516	-0.013	0.002	1.70E-10	454884	8.09E-05	36.797
rs117342986	T	C	0.026	0.037	0.007	1.80E-08	454884	6.91E-05	31.443

rs879620	T	C	0.613	0.024	0.002	5.70E-32	454884	2.76E-04	125.773
rs7498665	G	A	0.400	0.027	0.002	9.30E-39	454884	3.37E-04	153.344
rs862320	T	C	0.410	-0.023	0.002	2.20E-30	454884	2.61E-04	118.827
rs7774	A	C	0.310	0.015	0.002	1.50E-12	454884	1.01E-04	45.840
rs1017529	A	C	0.175	0.015	0.003	1.20E-08	454884	6.70E-05	30.470
rs2306593	T	C	0.488	-0.017	0.002	1.70E-17	454884	1.45E-04	65.821
rs11079849	T	C	0.329	-0.020	0.002	6.30E-22	454884	1.85E-04	84.170
rs7218014	C	T	0.197	0.019	0.003	7.80E-14	454884	1.12E-04	50.726
rs11150745	G	A	0.318	-0.021	0.002	2.30E-22	454884	1.89E-04	85.913
rs4790292	A	C	0.154	-0.026	0.003	6.90E-21	454884	1.76E-04	80.185
rs16966801	G	A	0.197	0.015	0.003	6.70E-09	454884	6.69E-05	30.433
rs62072006	C	A	0.145	0.016	0.003	2.30E-08	454884	6.25E-05	28.433
rs35867081	G	A	0.512	-0.015	0.002	2.00E-13	454884	1.08E-04	49.324
rs9674487	G	C	0.001	0.160	0.029	2.90E-08	454884	6.84E-05	31.108
rs118136827	T	G	0.281	-0.014	0.002	1.10E-09	454884	7.39E-05	33.632
rs8076669	C	T	0.562	0.014	0.002	5.90E-12	454884	9.43E-05	42.921
rs1320251	T	C	0.455	-0.018	0.002	1.50E-18	454884	1.55E-04	70.334
rs59237168	C	T	0.216	-0.016	0.002	8.20E-11	454884	8.41E-05	38.275
rs56161855	T	A	0.133	0.023	0.003	1.10E-14	454884	1.19E-04	54.141
rs1788808	G	A	0.495	-0.021	0.002	8.80E-25	454884	2.11E-04	95.906
rs559231	T	G	0.393	0.013	0.002	3.20E-10	454884	7.93E-05	36.092
rs6567160	C	T	0.233	0.054	0.002	2.50E-114	454884	1.03E-03	467.554
rs7232171	T	G	0.583	0.013	0.002	1.20E-10	454884	8.25E-05	37.553
rs1834144	A	C	0.373	-0.014	0.002	8.10E-12	454884	9.35E-05	42.529
rs512121	C	T	0.192	-0.016	0.003	6.70E-10	454884	7.63E-05	34.717
rs60764613	T	G	0.145	0.020	0.003	1.60E-12	454884	1.00E-04	45.684

rs784257	C	T	0.813	0.018	0.003	2.40E-12	454884	9.87E-05	44.900
rs9951619	G	T	0.767	0.015	0.002	2.90E-10	454884	8.01E-05	36.435
rs57636386	C	T	0.084	-0.041	0.004	1.00E-29	454884	2.57E-04	116.735
rs12956148	A	C	0.278	0.014	0.002	7.90E-10	454884	7.53E-05	34.252
rs10417386	C	T	0.696	0.012	0.002	1.30E-08	454884	6.45E-05	29.353
rs55714539	C	A	0.344	0.018	0.002	3.50E-17	454884	1.44E-04	65.307
rs8112818	G	A	0.400	-0.020	0.002	2.30E-23	454884	1.99E-04	90.322
rs12459368	G	A	0.268	-0.017	0.002	1.20E-14	454884	1.18E-04	53.741
rs12462975	A	G	0.330	0.019	0.002	8.50E-20	454884	1.67E-04	76.115
rs12977787	A	G	0.541	0.013	0.002	1.70E-11	454884	9.01E-05	40.988
rs56356382	C	T	0.192	-0.023	0.003	6.70E-19	454884	1.58E-04	71.986
rs56352336	C	T	0.155	-0.017	0.003	1.80E-09	454884	7.28E-05	33.111
rs7250833	T	C	0.289	0.014	0.002	7.10E-10	454884	7.58E-05	34.502
rs429358	C	T	0.154	-0.027	0.003	5.90E-22	454884	1.85E-04	84.102
rs10423928	A	T	0.194	-0.033	0.003	2.20E-40	454884	3.51E-04	159.794
rs3764625	G	T	0.588	-0.011	0.002	1.50E-08	454884	6.41E-05	29.149
rs73026725	A	C	0.154	-0.022	0.003	9.70E-16	454884	1.29E-04	58.577
rs7259070	C	T	0.596	0.022	0.002	3.80E-26	454884	2.27E-04	103.197
rs34481751	A	C	0.165	-0.019	0.003	2.20E-12	454884	1.01E-04	45.966
rs2153740	G	A	0.480	-0.011	0.002	1.20E-08	454884	6.55E-05	29.780
rs4456769	T	C	0.333	0.015	0.002	3.80E-12	454884	9.61E-05	43.707
rs2425816	A	G	0.415	0.012	0.002	7.90E-10	454884	7.53E-05	34.273
rs909892	A	G	0.135	-0.018	0.003	8.60E-10	454884	7.56E-05	34.372
rs73142879	T	C	0.192	-0.027	0.003	9.30E-27	454884	2.30E-04	104.658
rs55886426	G	C	0.053	-0.028	0.005	1.90E-09	454884	7.76E-05	35.279
rs6134916	T	C	0.493	-0.011	0.002	4.60E-08	454884	5.98E-05	27.199

rs1884897	G	A	0.627	0.020	0.002	1.70E-21	454884	1.82E-04	82.841
rs947088	T	G	0.718	0.013	0.002	9.80E-09	454884	6.59E-05	29.967
rs6023649	G	A	0.743	-0.014	0.002	1.50E-09	454884	7.50E-05	34.122
rs8132491	A	G	0.313	-0.016	0.002	2.10E-12	454884	1.04E-04	47.199
rs8134638	C	T	0.376	0.012	0.002	1.90E-09	454884	7.22E-05	32.834
rs17193211	T	C	0.067	-0.025	0.004	1.10E-09	454884	7.64E-05	34.772
rs394608	C	T	0.538	0.019	0.002	2.30E-20	454884	1.72E-04	78.085
rs406388	G	C	0.177	0.016	0.003	1.40E-09	454884	7.35E-05	33.457
rs5995843	G	A	0.346	-0.018	0.002	5.00E-17	454884	1.40E-04	63.902
rs28489620	A	G	0.290	-0.015	0.002	7.70E-12	454884	9.48E-05	43.137

Supplementary Table S4. Causal effect of *H. pylori* infection on the diagnosis of CHD.

Outcomes	nSNPs	OR	*P* value	Low_95%CI	High_95%CI
CHD (IEU)	2	0.991	0.842	0.904	1.078
CHD (Finn)	2	1.049	0.178	0.980	1.118
MI (Finn)	2	0.993	0.889	0.896	1.091
Angina pectoris (Finn)	2	1.105	0.023	1.019	1.191

SNPs, the number of SNPs used in analysis. The SNP rs10004195 and rs368433 were used if nSNP = 2. OR, the odds ratio; 95% CI, 95% confidence interval; CHD, coronary heart disease; MI, myocardial infarction.

Supplementary Table S5. Causal effect of *H. pylori* infection on the prognosis of CHD.

**Outcomes**	**nSNPs**	**OR**	***P* value**	**Low_95%CI**	**High_95%CI**
MACE (IEU)	2	0.999	0.391	0.997	1.001
MACE (Finn)	2	1.022	0.663	0.922	1.123
Heart arrhythmia	1	1.000	0.823	0.999	1.001
Heart attack	1	0.998	0.124	0.996	1.000
Stroke	1	0.999	0.525	0.998	1.001
Heart failure	2	1.000	0.741	0.999	1.001
Target HR achieved	1	0.994	0.252	0.983	1.005
Maximum HR	1	0.972	0.115	0.937	1.007
Cardiogenic death	2	1.035	0.558	0.919	1.151

SNPs, the number of SNPs used in analysis, the SNP rs10004195 was used if nSNP = 1. The SNP rs10004195 and rs368433 were used if nSNP = 2. OR, the odds ratio; 95% CI, 95% confidence interval; MACEs, major adverse cardiovascular events; Target HR achieved, reached target heart rate; Maximum HR, maximum heart rate during fitness test.

Supplementary Table S6. Causal effect of *H. pylori* infection on the pathogenic mechanism of CHD.

**Outcomes**	**nSNPs**	**β**	***P* value**	**Low_95%CI**	**High_95%CI**
FBG	2	0.006	0.511	-0.011	0.023
TG	2	0.005	0.409	-0.006	0.016
HDL	2	-0.006	0.788	-0.047	0.035
LDL	2	0.013	0.515	-0.026	0.051
BMI	2	0.022	0.001	0.008	0.035
Vitamin C	1	-0.002	0.318	-0.006	0.002
Vitamin D	1	0.000	0.775	-0.003	0.002
Vitamin B12	1	0.008	0.685	-0.029	0.044
Interleukin-18	2	0.022	0.493	-0.041	0.086
Interleukin-6	2	-0.041	0.294	-0.117	0.035
Interleukin-8	2	0.017	0.645	-0.055	0.089
Interleukin-4	2	-0.066	0.497	-0.258	0.125
Interleukin-10	1	-0.079	0.429	-0.276	0.117
TNF-α	1	0.020	0.893	-0.275	0.316

SNPs, the number of SNPs used in analysis, the SNP rs10004195 was used if nSNP = 1. The SNP rs10004195 and rs368433 were used if nSNP = 2. OR, the odds ratio; 95% CI, 95% confidence interval; FBG, fasting blood glucose; TG, triglycerides; HDL-C, high-density lipoprotein cholesterol; LDL-C, low-density lipoprotein cholesterol; BMI, body mass index; TNF-α, tumor necrosis factor-α.

Supplementary Table S7. Causal effect of BMI on the diagnosis of CHD.

**Outcomes**	**nSNPs**	**OR**	***P* value**	**Low_95%CI**	**High_95%CI**
Angina pectoris (MR Egger)	424	1.118	4.25E-01	0.844	1.392
Angina pectoris (Weighted median)	424	1.362	2.02E-04	1.199	1.525
Angina pectoris (IVW)	424	1.275	2.47E-06	1.174	1.377
CHD-IEU (MR Egger)	419	1.692	2.09E-07	1.497	1.888
CHD-IEU (Weighted median)	419	1.501	2.49E-12	1.387	1.615
CHD-IEU (IVW)	419	1.493	5.20E-28	1.421	1.565
MI (MR Egger)	424	1.317	7.50E-02	1.015	1.619
MI (Weighted median)	424	1.472	1.63E-05	1.296	1.648
MI(IVW)	424	1.320	1.08E-06	1.208	1.432
CHD-Finn (MR Egger)	424	1.233	5.52E-02	1.020	1.447
CHD-Finn (Weighted median)	424	1.444	2.27E-09	1.324	1.565
CHD-Finn (IVW)	424	1.282	6.53E-10	1.203	1.361

SNPs, the number of SNPs used in analysis. OR, the odds ratio; 95% CI, 95% confidence interval; CHD, coronary heart disease; MI, myocardial infarction.

Supplementary Table S8. Causal effect of CHD on *H. pylori* infection.

Exposure	nSNPs	OR	*P* value	Low_95%CI	High_95%CI
Angina pectoris (MR Egger)	20	1.167	0.219	0.929	1.405
Angina pectoris (Weighted median)	20	1.068	0.334	0.934	1.203
Angina pectoris (IVW)	20	1.067	0.202	0.967	1.167
CHD-IEU (MR Egger)	38	1.116	0.359	0.885	1.346
CHD-IEU (Weighted median)	38	1.115	0.138	0.971	1.258
CHD-IEU (IVW)	38	1.091	0.091	0.990	1.191
MI (MR Egger)	11	1.272	0.153	0.970	1.575
MI (Weighted median)	11	1.125	0.139	0.969	1.282
MI (IVW)	11	1.110	0.063	1.000	1.220
CHD-Finn (MR Egger)	25	1.164	0.242	0.916	1.412
CHD-Finn (Weighted median)	25	1.095	0.258	0.938	1.252
CHD-Finn (IVW)	25	1.076	0.183	0.968	1.184

SNPs, the number of SNPs used in analysis, OR, the odds ratio; 95% CI, 95% confidence interval; CHD, coronary heart disease; MI, myocardial infarction.
